# A worldwide itinerary of research ethics in science for a better social responsibility and justice: a bibliometric analysis and review

**DOI:** 10.3389/frma.2025.1504937

**Published:** 2025-02-11

**Authors:** Ingrid Sonya Mawussi Adjovi

**Affiliations:** Ethics and Social Responsibility Research Unit (UR-ERS), Research Laboratory on Innovation for Agricultural Development (LRIDA), University of Parakou, Parakou, Benin

**Keywords:** ethics in research, university, community involvement, social equity, bibliometric mapping, review

## Abstract

This study provides a comprehensive overview of research ethics in science using an approach that combine bibliometric analysis and systematic review. The importance of ethical conduct in scientific research to maintain integrity, credibility, and societal relevance has been highlighted. The findings revealed a growing awareness of ethical issues, as evidenced by the development of numerous guidelines, codes of conduct, and oversight institutions. However, significant challenges persist, including the lack of standardized approaches for detecting misconduct, limited understanding of the factors contributing to unethical behavior, and unclear definitions of ethical violations. To address these issues, this study recommends promoting transparency and data sharing, enhancing education, and training programs, establishing robust mechanisms to identify and address misconduct, and encouraging collaborative research and open science practices. This study emphasizes the need for a collaborative approach to restore public confidence in science, protect its positive impact, and effectively address global challenges, while upholding the principles of social responsibility and justice. This comprehensive approach is crucial for maintaining research credibility, conserving resources, and safeguarding both the research participants and the public.

## 1 Introduction

### 1.1 Importance of research ethics and main focal points

Research ethics in science are important for the quality of scientific production. This significantly affects the integrity, credibility, and societal importance of scientific research, academic projects, and innovation (Armond et al., 2021). As more scholars explore different fields of study, the importance of maintaining ethical principles becomes paramount, not only for the advancement of scientific knowledge (Ambroz and Bukovec, 2015), but also for the establishment of social responsibility and social justice (Martínez-Valdivia et al., 2020) in many countries.

Research ethics focuses on guaranteeing research integrity, honesty, and responsibility (Lau, 2021). Informed consent, data manipulation, plagiarism, and conflicts of interest are ethical issues. Unethical practices can damage research reliability and public trust in science, and harm individuals or communities (Sivasubramaniam et al., 2021). Adhering to rigorous research ethics is essential for preserving scientific and social responsibilities.

### 1.2 Origin of the problem and urgency of addressing it

The origin of this problem can be traced to several factors. The “publish or perish” culture in academia can create intense competition among researchers to quickly produce significant results (Amutuhaire, 2022; Becker and Lukka, 2022). This pressure may lead to unethical practices such as data manipulation or selective reporting to present more favorable outcomes. Financial incentives are another important factor to consider. Research funding and financial rewards associated with successful publications can create conflicts of interest, influencing researchers to prioritize certain results over others, or engage in questionable research practices (Ghose et al., 2021; Jay et al., 2019; Seifert et al., 2023). Occasionally, a lack of training is the main ethical problem in research. Some researchers may not receive adequate training or education in research ethics, leading to an unawareness or misunderstanding of the ethical principles and guidelines guidelines (Wilson et al., 2018; Yeoh et al., 2017). Faced with this worrying situation, it is crucial to examine the factors contributing to the crisis and to explore potential solutions to strengthen the reliability of scientific research.

There has been a replication crisis in many disciplines. The growing concern about the inability to replicate many scientific studies has shed light on issues of research misconduct, inadequate methodology, and publication bias, raising questions regarding the reliability of research findings (Boulesteix et al., 2020; Cantley, 2023; Cooper, 2018; Hope et al., 2021; Ryan and Tipu, 2022).

It has also been proven (Sorokowski et al., 2019) that one of the most significant problems faced by modern scientific publishing systems is the pressure to publish positive results, which is a major drawback. In some cases, institutions or journals may have insufficient oversight to detect and address unethical behavior (Bain et al., 2018; Malviya, 2012; Resnik, 2023; Thorogood and Knoppers, 2017), allowing misconduct to go unnoticed or unpunished. According to some authors, the main factors explaining the lack of research ethics are related to data sharing and transparency. Beyond these factors, the emergence of new ethical challenges in emerging fields could be problematic. Fields such as human resources (Edwards et al., 2022), artificial intelligence (de Zárate Alcarazo, 2022), and Tissue Engineering (TE) for Regenerative Medicine (RM) (De Kanter et al., 2023) may present new ethical dilemmas that researchers and regulators may find difficult to resolve.

### 1.3 Strategies and solutions for addressing research ethics challenges

To solve these problems and overcome the challenges of research ethics, scientists have established rigorous peer review processes to evaluate research proposals and publications (Malviya, 2012), create and share ethical codes and guidelines for reference (Santos et al., 2017), promote transparency and data sharing to prevent data manipulation and facilitate replication (Boulesteix et al., 2020; Hassan et al., 2022), obtain informed consent and ethical approval to protect research participants (Afolabi, 2012; Moore and Savage, 2002), and provide education and training programs to promote academic integrity and ethical behavior. It also established mechanisms to identify and address research misconduct, such as plagiarism and fabrication (Agrahari and Sharma, 2017; Langlais, 2006; Laskar, 2017; Mohammed and Abdelsalam, 2022; Reisig et al., 2020), and encouraged collaborative research and interdisciplinary partnerships (Fairhall et al., 2016; Nyström et al., 2018) to bring diverse views, reduce ethical bloodspots, and practice open science to encourage reproducibility and transparency (Wolfram et al., 2020), among other steps. Additionally, reporting negative or inconclusive results prevents publication bias (Van Aert and Niemeyer, 2022; Lin and Chu, 2018) and creates balanced scientific literature. Leadership and institutional commitment to research integrity are essential for prioritizing and rewarding ethical conduct.

The urgency of addressing the research ethics issue lies in its potential consequences, including misdirection of policymakers, hindrance of scientific progress, and waste of resources. Such misconduct also harms public trust in science and can endanger society's wellbeing (Goldenberg, 2022; Huber et al., 2019; Intemann, 2023). Immediate action is necessary to maintain research credibility, conserve resources, and safeguard both the research participants and the public. A collaborative approach is crucial for restoring confidence in science, protecting its positive impact, and addressing global challenges.

### 1.4. Current gaps and new challenges in bibliometric analysis and systematic review

The current state of knowledge regarding research ethics in scientific publications shows a considerable awareness of the major role of ethical conduct in the research process (Tormo-Carbó et al., 2018). Numerous institutions, guidelines, and codes of conduct have been developed to address the ethical concerns in various research domains (Franzke, 2022; Larsson, 2020; Laws and Utne, 2019).

However, several gaps and shortcomings remain unaddressed. Methodologically, there is a lack of standardized and comprehensive approaches for detecting research misconduct (Girgin et al., 2022; Hawkins et al., 2018; Matosas-López and Cuevas-Molano, 2022), leading to difficulties in identifying and preventing unethical behavior. Theoretical gaps exist in understanding the underlying factors that contribute to research misconduct (Dal-Ré et al., 2020; Golden et al., 2023; Haven and van Woudenberg, 2021; Horbach et al., 2020; Rodrigues et al., 2023) and how to foster a culture of research integrity effectively. Conceptually, there is a need for clearer definitions and classifications of the different types of research ethics violations to facilitate better reporting and analysis (Shaw, 2019). Contextually, challenges arise from the evolving landscape of research, such as emerging fields and the influence of technology, which may create new ethical dilemmas that are not adequately addressed in the existing guidelines (de Zárate Alcarazo, 2022; Edwards et al., 2022; De Kanter et al., 2023). Moreover, the universal enforcement of research ethics guidelines is lacking, leading to inconsistent practices and accountability across research communities and institutions (Ataullahjan et al., 2022; Mills et al., 2020). Ethical considerations in interdisciplinary research and cross-cultural studies require more attention to ensure that ethical principles are applied appropriately across diverse contexts (Bonde et al., 2016; Kwame and Petrucka, 2023; Quester and Simpson, 1998; Rawwas et al., 2005). Furthermore, there is a need to promote open science practices and data sharing to enhance research transparency and to reduce the risk of publication bias (Wolfram et al., 2020). Addressing these gaps requires collaborative efforts from researchers, institutions, policymakers, and funding agencies to strengthen the foundation of research ethics, foster a culture of integrity, and uphold responsible conduct within scientific communities.

Previous bibliometric analyses and systematic reviews on research ethics in scientific publications have revealed several inadequacies. They often focus on narrow subfields, which leads to a limited perspective. Incomplete data coverage and inadequate contextual information have hindered a comprehensive understanding. Overreliance on quantitative metrics has overlooked the qualitative aspects of the ethical challenges. However, emerging ethical issues and cultural contexts have not been explored sufficiently. Neglecting open science practices has hindered holistic assessment of research integrity. Addressing these limitations is crucial for a more robust understanding of research ethics and fostering responsible scientific practices. To overcome these limitations, this study aims to emphasize social responsibility and justice in the application of research ethics principles across various fields of specialization. This study was structured to address the following research questions:

- How is the evidence base distributed concerning the total number of scientific documents, their research fields, annual production by subject area, and the most relevant authors by subject area?
- How do you explain the emergence of these themes in a particular field?
- Why are ethics and social responsibility more widely debated in some disciplines than in others?
- What explains the profusion of the debate on research ethics in some countries but not in others?

To enhance the systematic management of the literature corpus, this study restricted the bibliographic search to the Scopus database. The resultant dataset was exported in the CSV format to facilitate subsequent data processing and analysis procedures.

## 2 Research methodology

### 2.1 Research design, data collection, and search strategies

As a mixed article, this study utilized Bibliometrix R, a potent programming language for statistical analysis and data visualization, to conduct a bibliometric analysis. Bibliometrix offers a comprehensive set of functions to analyse, visualize, and explore vast bibliographic data, including descriptive statistics, co-authorship network analysis, keyword analysis, and citation analysis. This provides valuable insights into the patterns and trends within their research domains. To show the actual state of the research ethics debate in various fields and its gaps, this research applied the Problem, Intervention and Outcomes (PIO) approach for a short systematic review. Literature recommends the application of formal processes such as the PICO model (Problem, Intervention, Comparator and Outcomes) to carry out systematic reviews and maps to effectively synthesize behavioral (Berger-Tal et al., 2019; Considine et al., 2017; Pollock and Berge, 2018; Verschuren and Doorewaard, 2010). The PIO method is a variant of PICO that does not consider the comparator and has been used in several studies.

P: Research studies focusing on research ethics, frameworks, or principles applied in various scientific or societal contexts.

I: Application, utilization, or adherence to ethical principles, research integrity, or methodologies aligned with ethical guidelines. O: Impact on social equity, justice, sustainability, social responsibility, and inclusivity.

The applied research strings were as follows:

“Research ethics” OR “Ethical considerations” OR “Ethical principles” OR “Ethical guidelines” OR “Moral standards” OR “Research integrity” OR “Ethical framework” OR “Responsible conduct of research” OR “Ethical protocol” OR “Ethical standards” OR bioethics OR “ethics in research” OR “ethic^*^ in science” OR “morality in science” OR “ethic^*^ principles” OR “methodologic^*^ values” OR “virtue in research” OR “scientific^*^ integrity” OR “scientific conscience” OR “honesty in science”)

AND

(use OR conducting OR respect OR us^*^ OR utiliz^*^ OR employ^*^ OR apply^*^ OR applicat^*^ OR “Mak^*^ use of” OR exercis^*^ OR “Resort to” OR “Call upon” OR adopt^*^ ”” OR practic^*^)

AND

(“Scientific responsibility” OR “Corporate social responsibility” OR csr OR “Ethical responsibility” OR “Social accountability” OR “Civic responsibility” OR “Community responsibility” OR sustainab^*^ OR “Public interest” OR “Environmental stewardship” OR “Social consciousness” OR “Community engagement” OR ”” OR “Distributive justice” OR “Economic justice” OR “Racial justice” OR “Environmental justice” OR “Gender justice” OR moral OR “Human rights” OR equity OR “Social equality” OR fairness OR inclusivity).

The document types included articles, reviews, and conference papers published in French and English.

The search for scientific publications was conducted on 27/08/2023, and the results were not updated. As final dataset, this research obtained a total of 3,128 documents published between 1965 and 2023. The metadata for each publication included citation and bibliographical information, abstracts, keywords, funding details, and other relevant information.

To ensure consistency of the data, I limited the search to English and French publications. Furthermore, I have focused on peer-reviewed journal articles, reviews, and conference papers to ensure credibility and reliability of the sources. The following section provides a detailed description of this process.

### 2.2 Data processing, bibliometric, and systematic analysis

The process began with the formulation of a search equation in the SCOPUS database. The application of this equation yielded an initial database of 3,615 documents. This was followed by direct screening of titles and abstracts in SCOPUS to refine the selection of relevant documents. At the end of this exercise, a database of 3,128 documents was obtained. Once this screening was completed, the data were extracted from SCOPUS, marking the end of the data collection phase. These data were then processed using two parallel approaches during the analysis phase. On the one hand, the Bibliometrix package is launched and loaded to enable general bibliometric analysis via the Biblioshiny interface. Data were imported into Microsoft Excel for comparative analysis by thematic area.

These two approaches converge in the final phase of data visualization. The process culminates in a comprehensive synthesis comprising several elements: a general overview of the literature, analysis of sources, review of key authors, study of documents, geographical mapping of research, and representation of the conceptual intellectual structure that highlights current debates and emerging lines of research.

**Figure 1:**
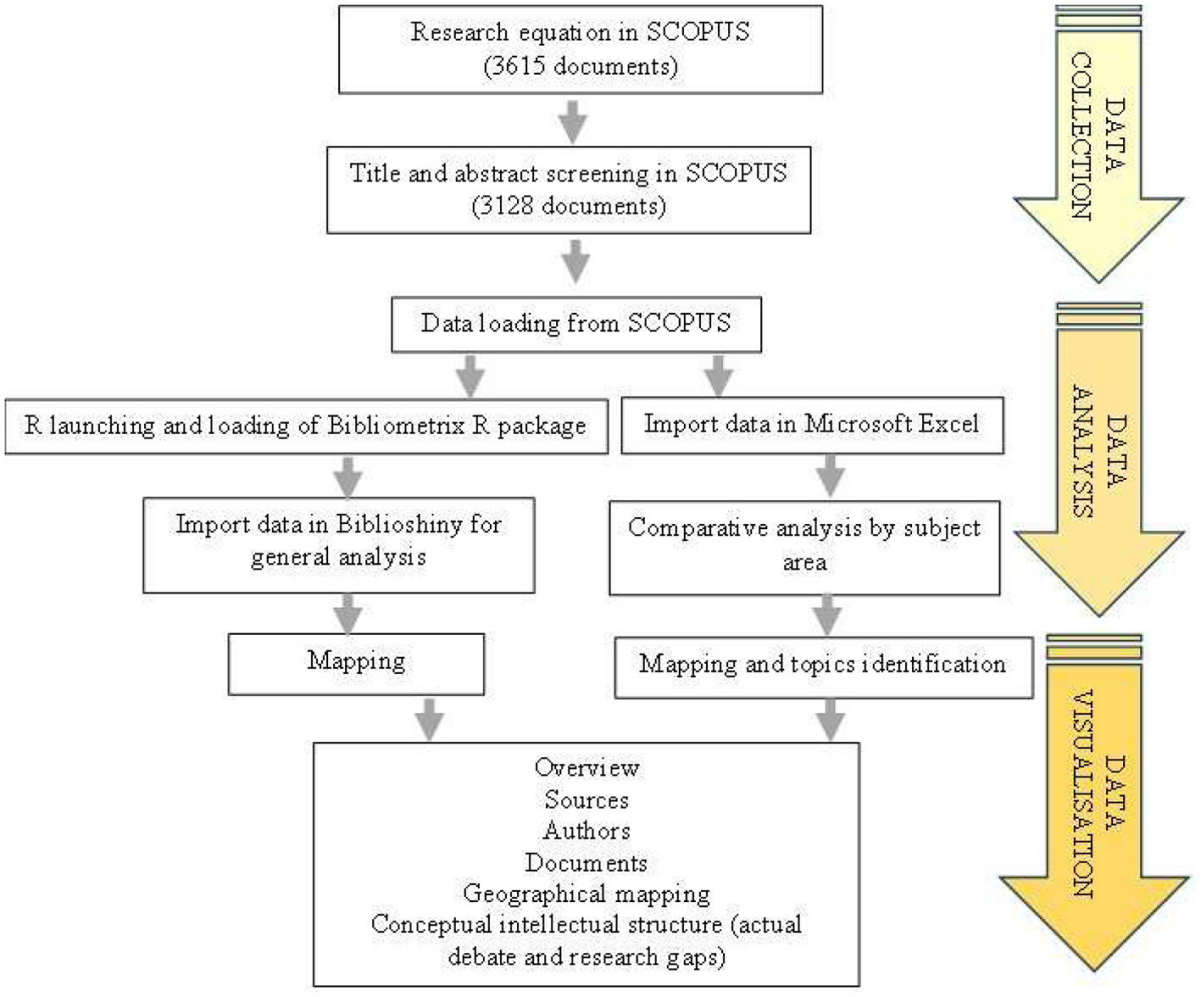
Data gathering and data analysis process.

The results are described in the form of frequency histograms, bar charts, curves, maps, and tables, which were analyzed and discussed. The journal impact factors presented in the results come from information available on journal websites in the Web of Science. They show the impact factors for each journal over the last 5 years. To highlight the current debates and gaps in the research theme, the Litmaps visualization tool was used to identify and analyse key publications. The process of data processing and bibliometric and systematic analyses are shown in Figure 1.

This mixed methodology combines the advantages of a quantitative bibliometric analysis with those of a qualitative systematic review, offering a richer and more nuanced understanding of the literature.

## 3 Results

This section presents the analysis outcomes, encompassing patterns in article publications, prominent journals, influential countries/regions, authors, scientific collaborations, and clustering.

### 3.1 Bibliometrics descriptive statistics

This research analyzed the existing knowledge of research ethics for better social responsibility and social justice through a large panel of subject areas (Table 1). A total of 3,128 documents (2470 articles, 157 conference papers, and 501 reviews) were published between 1965 and 2023, spanning a period of 58 years. These publications contain 115,700 references, and each document is cited at an average of 17.36 times, indicating that they are well-regarded in academic circles.

**Table 1:** Descriptive statistics.

**Description**	**Results**
**Main information about data**	
Timespan	1965:2023
Sources (Journals, Books, etc)	1,516
Documents	3,128
Annual growth rate %	9.31
Document average age	10.8
Average citations per doc	17.36
References	115,700
**Document contents**	
Keywords plus (ID)	7,243
Author's keywords (DE)	6,503
**Authors**	
Authors	8,461
Authors of single-authored docs	1,079
**Authors collaboration**	
Single-authored docs	1,169
Co-Authors per Doc	3.04
International co-authorships %	16.75
**Document types**	
Article	2,470
conference paper	157
Review	501

The keywords generated automatically by an algorithm from the reference citation in the documents slightly outnumber the authors' keywords (7,243–6,503), suggesting that they are more informative than the authors' keywords (63, 64).

The need to consider ethics throughout the scientific research process has led to several collaborative scientific contributions. This explains the average proportion of co-authors per document (3.04) and the international nature of the research teams (16.75%).

#### 3.1.1 Distribution by main subject area

**Figure 2:**
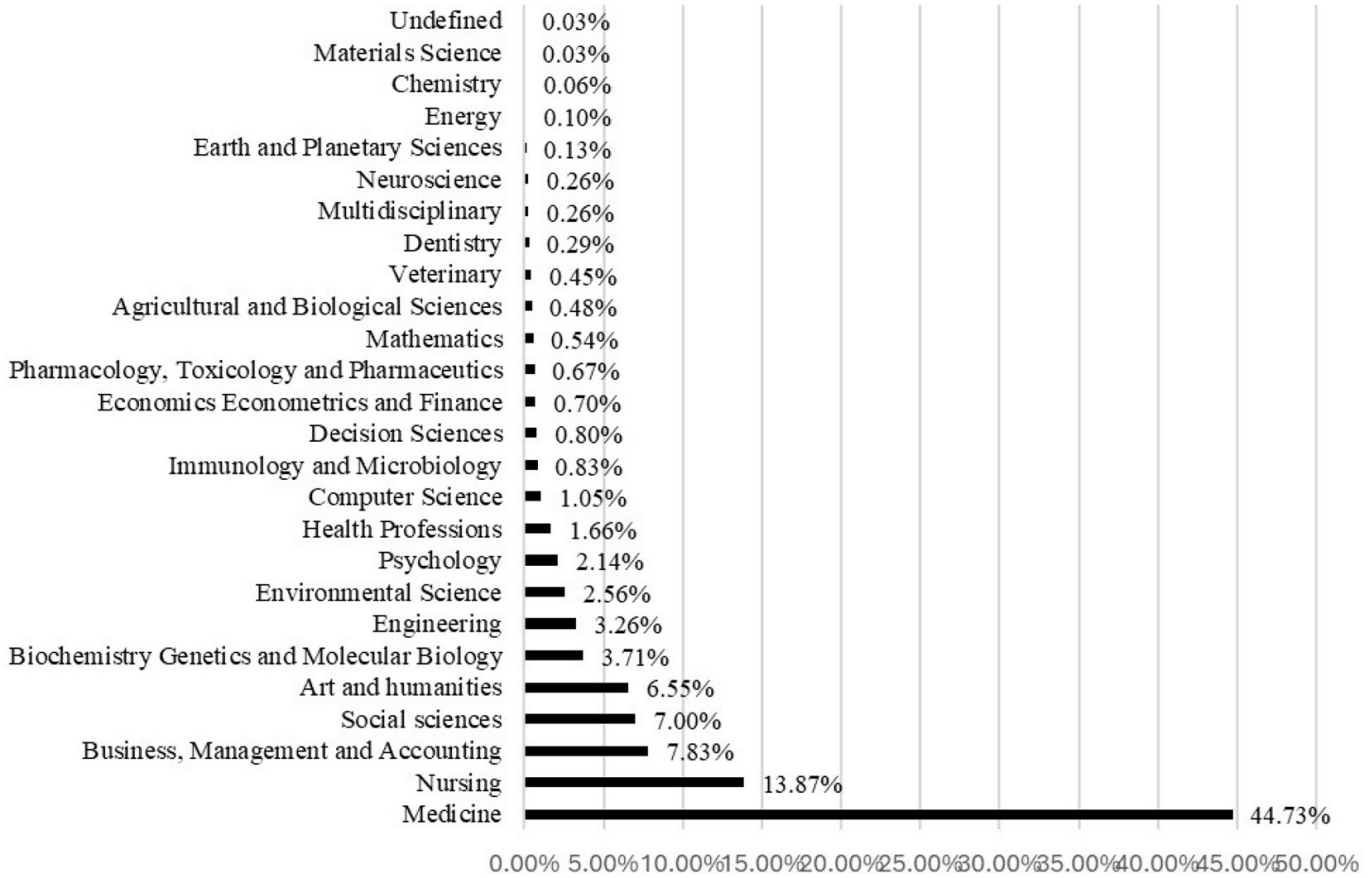
Distribution by document subject area.

The findings showed that this collection of 3,128 documents is related to many scientific disciplines. Figure 2 shows that 28 subject areas were concerned with scientific documents related to ethics in the research. The main research areas were medicine (44.73%), nursing (13.87%), Business Management and Accounting (7.83), social sciences (7.00%), arts and humanities (6.55%), and Biochemistry Genetics and Molecular Biology (3.71%).

#### 3.1.2 Annual production of research

##### 3.1.2.1 Global research annual production

**Figure 3:**
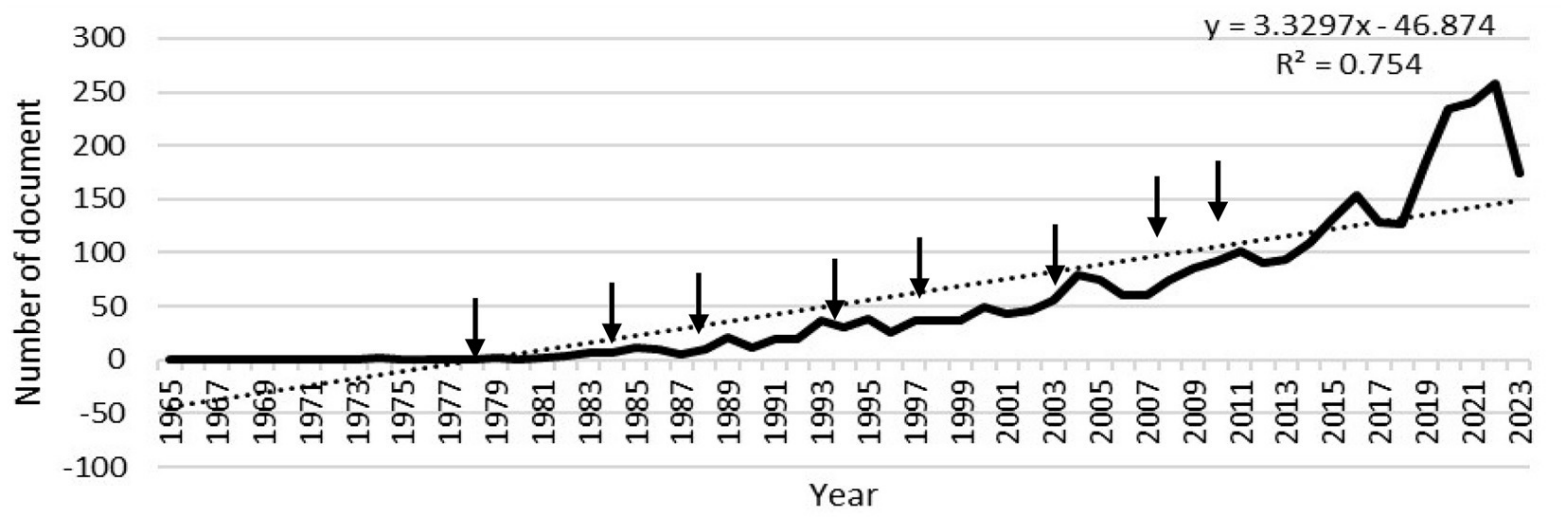
Annual global scientific production by year.

The progress from 1965 to 2023 in global scientific production by year is shown in Figure 3. The curve showing the evolution of scientific publications on research ethics indicates that the increase in the number of publications can be broadly divided into three periods: 1965 to 1985, 1985 to 2003, and after 2003.

During the first period, from 1965 to 1985, publication activity remained consistently low, with minimal variation, and very few articles were published annually. The second period, spanning 1985 to 2003, exhibits a slight but steady increase in publication numbers, marking the beginning of a growing interest in research ethics. The third period, starting after 2003, demonstrated a marked acceleration in publication activity, characterized by a steeper upward trend. This final period is particularly notable for its huge increase in publications, culminating in significant peaks exceeding 200 articles between 2020–2023, though with noticeable fluctuations. The overall trend is captured by a regression line (*y* = 3.3297*x* – 46.874) with an *R*^2^ value of 0.754, suggesting that time explains ~75.4% of the variation in publication numbers. This three-phase pattern reflects the evolving importance of research ethics in scientific discourse from a niche topic to a major concern in the scientific community. The most recent period particularly highlights contemporary emphasis on research ethics, driven by increased awareness of ethical considerations in scientific research and stricter publication requirements.

Even if Scopus was created not so far from 10 November 2004 by Elsevier, a Dutch academic publishing company, this scientific abstract and citation database covers many disciplines in a temporary coverage beginning in 1788 (Wikimedia Foundation, 2024).

##### 3.1.2.2. Annual production of research by subject topic and the emergence of research ethics in certain fields

**Figure 4:**
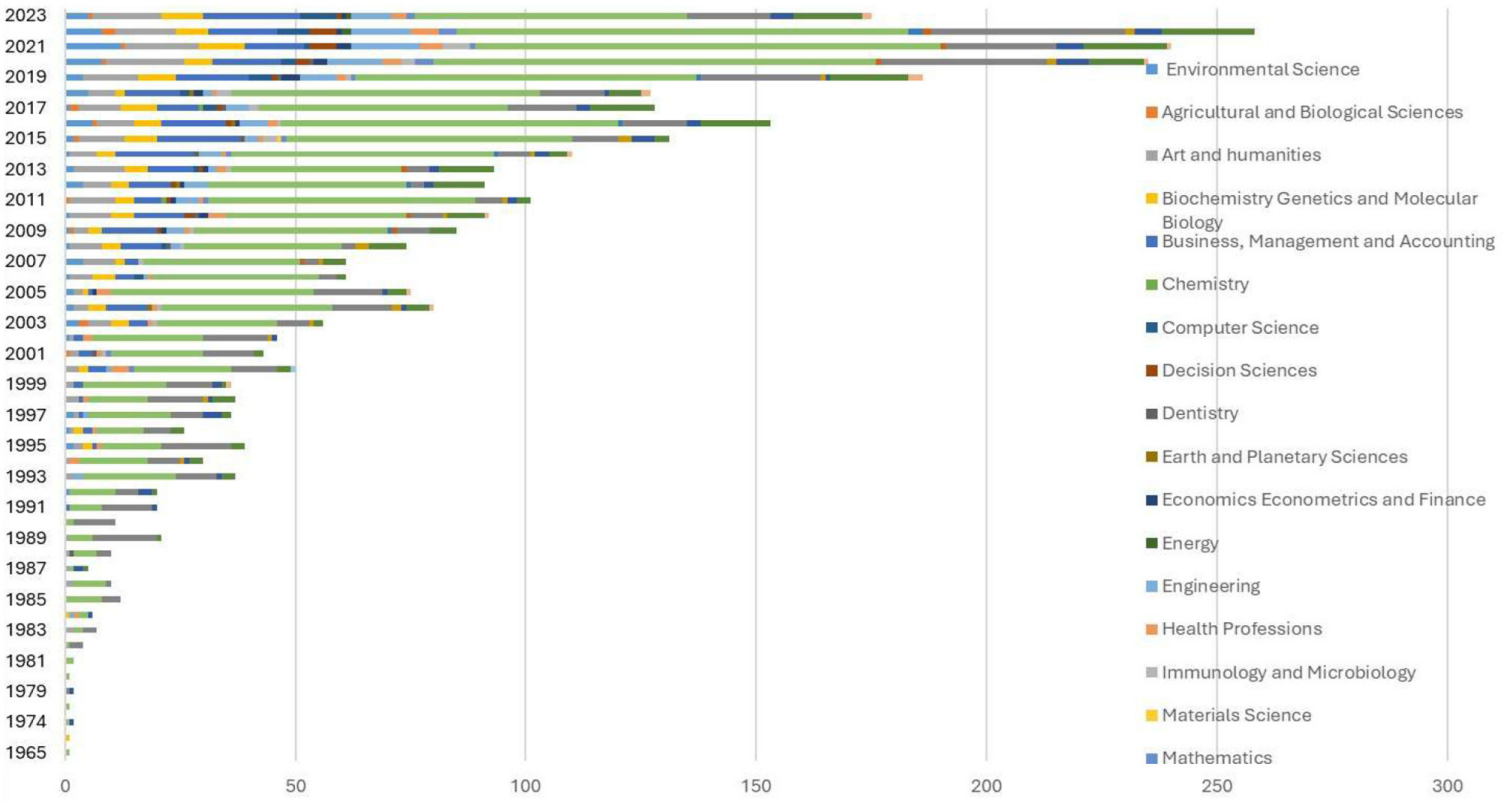
Annual scientific production by year and subject area.

The annual scientific output for each specialty was similar. The three main periods were identified for overall production. The Figure 4 bellow presents the annual scientific production by year and subject area since 1965.

The first scientific publications dealing with research ethics were in the field of medicine and nursing (1965–1985). Since 1987, dentistry has joined other publications in the field of health science. The first work on research ethics in psychology, the humanities, and social sciences was published in 1993. The findings show that the first publication on research ethics in the Agricultural and Biological Sciences dates to 2001. The first publication in Earth and Planetary Sciences was published in 2004. In 2005, the fields of economics and finance obtained their first publications. The first publication in the field of Computer Science was in 2006.

From 2007 onwards, a wide range of new fields of knowledge began to be published on the subject of research ethics, including Environmental Science, Art and Humanities, Biochemistry Genetics and Molecular Biology, Business, Chemistry, Decision Sciences, Energy, Engineering, Health Professions, Immunology and Microbiology, Mathematics, Multidisciplinary, Neuroscience, Pharmacology Toxicology and Pharmaceutics, and Veterinary and Undefined fields.

However, the field with the most recent publications on research ethics was Materials Science in 2015.

Generally speaking, publications in medicine and nursing dominate over the period from 1965 to 2023, except in the years 2007 to 2016, when, although medicine retained the lead, publications in management and accounting outnumbered those in nursing, which was second place.

#### 3.1.3 Most relevant authors

##### 3.1.3.1 Global relevant authors

**Figure 5:**
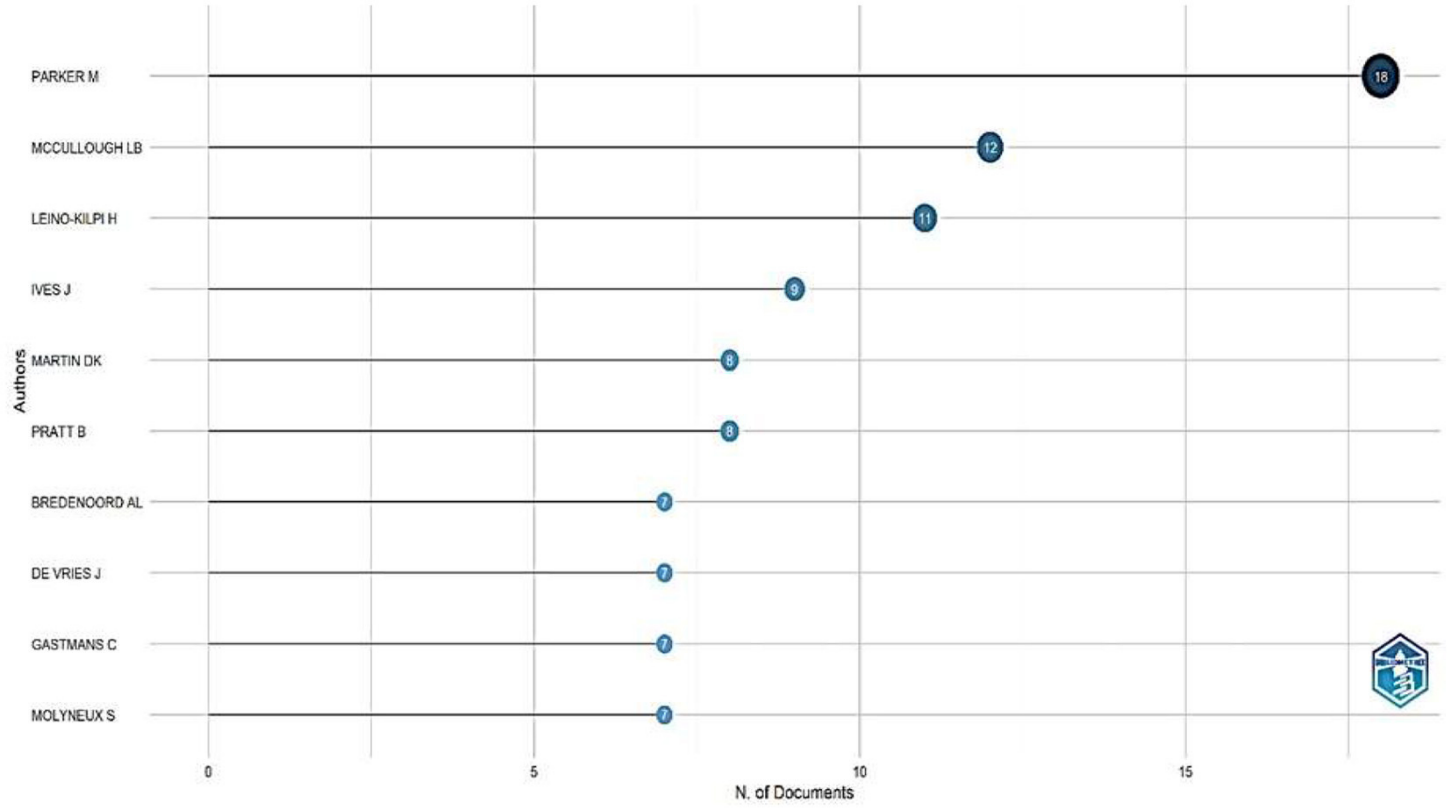
Most productive authors.

The results related to the 10 top authors (by the number of their productivity) conducting research on research ethics in different disciplines in science (Figure 5) showed that Parker M. had 18 publications, McCullough L.B. had 12 scientific articles, Leino-Kilpi H published 11 documents, Ives J. had nine papers, and Martin D.K. published eight articles. Other relevant authors include Pratt B. (8), Bredenoord A.L. (7), De Vries J, (7) Gastmans C. (7), and Molyneux S. (7).

##### 3.1.3.2 Most relevant authors by subject area

**Figure 6:**
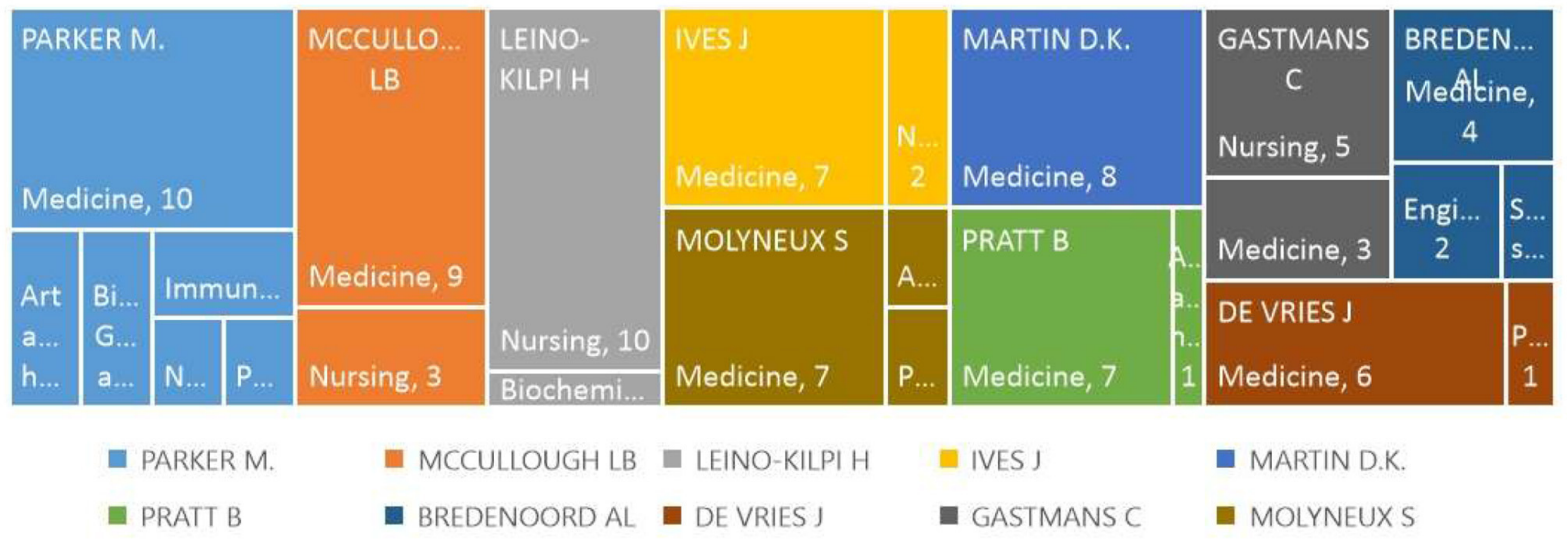
Most productive authors by subject area.

Figure 6 shows the 10 most productive authors in this field. These articles were published in various journals. Of the 97 publications, 61 were in the field of medicine, 21 in nursing, and four in Art and the Humanities; three publications were in Psychology and Biochemistry Genetics and Molecular Biology, and two in Engineering, Immunology and Microbiology. Only one document has been published in the social sciences. Each author publishes at least two specialist fields, except for Martin, who has only been published in medicine. There is a transdisciplinary approach to research ethics in the scientific literature. None of the most influential authors in this area of research are in the field of agronomic science. This finding suggests that questions related to the ethics of scientific research in this discipline are new.

#### 3.1.4 Most relevant institutions

The results obtained present 1,516 sources of publications of works collected from the Scopus database. The following table lists the sources of at least10 articles in the database.

Journals such as Nursing Ethics, Journal of Medical Ethics, BMC Medical Ethics, Bioethics, Science and Engineering Ethics, BMJ Open, Journal of Business Ethics, Social Science And Medicine, Developing World Bioethics, Journal of Advanced Nursing, Journal of Medicine and Philosophy (United Kingdom), Kennedy Institute of Ethics Journal, Journal of Bioethical Inquiry, Journal of Medicine And Philosophy, Medicine Health Care And Philosophy, Theoretical Medicine, and Bioethics are very popular with authors and have at least 20 articles published in the database. Among these sources of publications, the most influential, according to the 2023 impact factor ranking, is as follows in Table 2.

**Table 2:** The main institutions by number of articles and the 5-years impact factor.

**Sources**	**Articles**	**5-year impact factor**
Nursing Ethics	195	3.9
Journal of Medical Ethics	99	3.3
BMC Medical Ethics	84	3.5
Bioethics	71	2.01
Science and Engineering Ethics	57	3.5
BMJ Open	50	2.69
Journal of Business Ethics	38	8.1
Social Science and Medicine	34	4.42
Developing World Bioethics	31	1.95
Journal of Advanced Nursing	30	2.99
Journal of Medicine and Philosophy (United Kingdom)	28	NF
Kennedy Institute of Ethics Journal	28	1.9
Journal of Bioethical Inquiry	23	2.1
Journal of Medicine and Philosophy	22	1.28
Medicine, Health Care and Philosophy	21	2.0
Theoretical Medicine and Bioethics	21	1.6
Health Care Analysis	14	2.2
Hastings Center Report	13	4.29
Cambridge Quarterly of Healthcare Ethics	12	1.35
Medicine and Law	12	1.20
American Psychologist	11	10.75
ASEE Annual Conference And Exposition, Conference Proceedings	11	NF
International Journal of Environmental Research and Public Health	11	3.09
Journal of Agricultural and Environmental Ethics	11	2.2
Academic Medicine	10	7.4
Accountability in Research	10	2.42
Christian Bioethics	10	0.17
Medicine, Health Care, and Philosophy	10	2
Monash Bioethics Review	10	1.6
Perspectives in Biology and Medicine	10	0.92

NF, not found.

#### 3.1.5 Proliferation of research ethics publications by country

**Figure 7:**
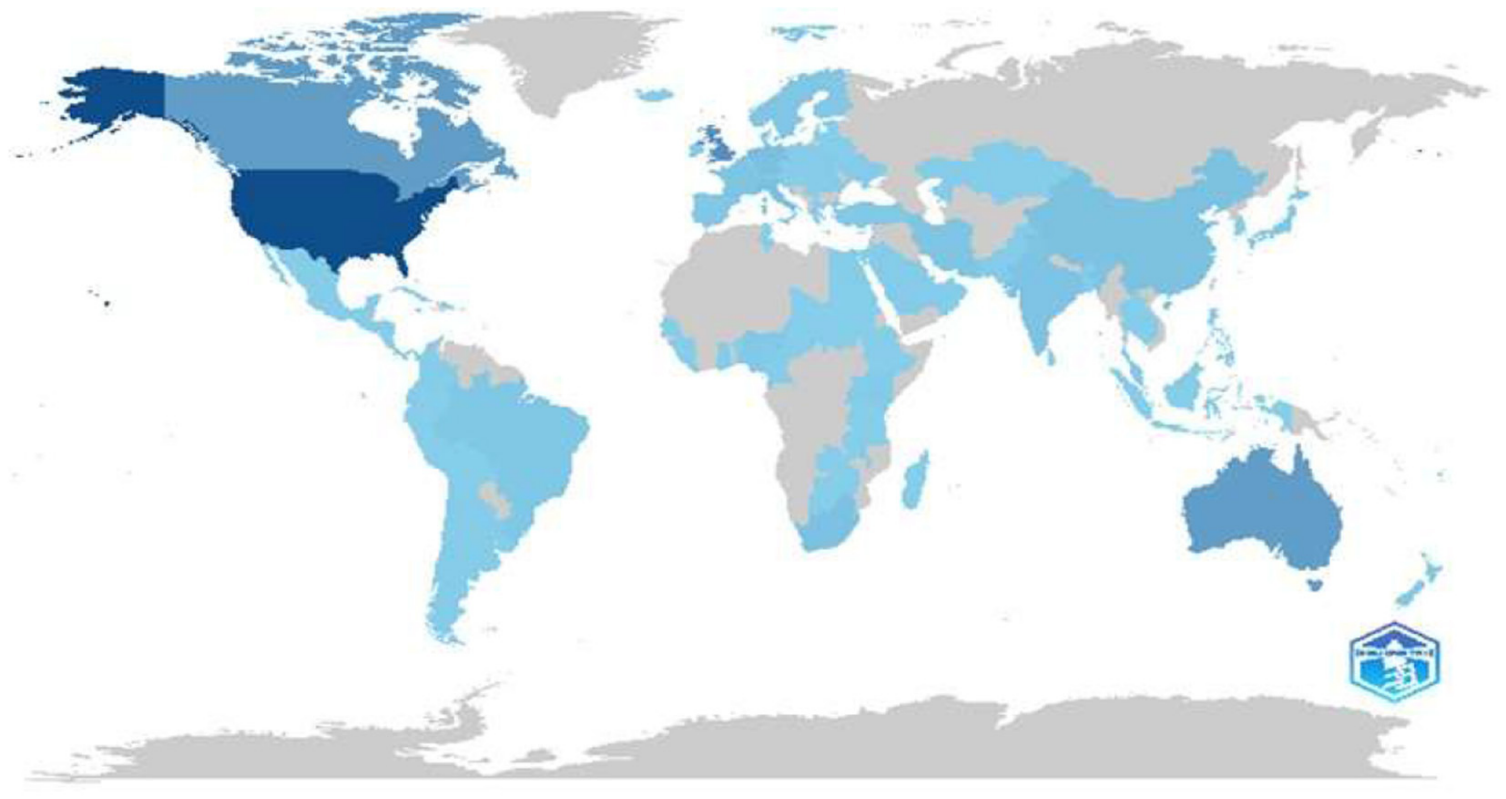
Productivity by continent.

The world map (Figure 7) illustrates the global distribution of scientific productivity, focusing on research ethics. The United States dominates the field with the highest output, followed by significant contributions from Australia and Canada. Europe and parts of Asia demonstrated moderate levels of research ethics publications, while South America showed lighter engagement. The map reveals a notable gap in research ethics output across Africa and parts of Asia, highlighting potential disparities in research capacity or documentation in these regions. This visualization effectively demonstrates the geographical imbalance in research ethics publications worldwide.

### 3.2 Main debate topics and gaps

Scientists and professionals frequently discuss research ethics. Table 3 below presents the distribution of the dataset publications across the major topics, offering an insight into the scope and focus of the ethical debates. It highlights three main categories of topics: ethical imperatives, medical research ethics, and methodological innovation. Medical research ethics dominates scientific discourse (1,158 publications). Key areas of focus include participant safety in clinical trials (70 publications), moral dimensions of medical ethics (61 publications) with some concern about organ transplants and euthanasia and randomized clinical trials (21 publications). Methodological innovation (615 publications) prioritize debate about participatory research (122 publications and ethical responsibility (397 publications). Scientists discuss ethical imperatives (483 publications) especially related to adolescent research ethics. These topics reflect the global and complex nature of ethical challenges in science. Emerging issues such as artificial intelligence and big data also highlight the growing importance of addressing technological advancements in research ethics.

**Table 3:** Contemporary trends in scientific debate.

**Actual debate related to research ethics**	**Level 1 sub-topic**	**Number of publications**	**Level 2 sub-topic**	**Number of publications**	**Level 3 sub-topic**	**Number of publications**
	Ethical Imperatives	483	Historical context	5	Nazi Atrocities	4
			Moral dimension	30	Emergency Medicine Regulations	3
			Parental consent issues	4		
			Adolescent research ethics	92		
	Medical Research Ethics	1158	Randomized clinical trials	21	Suboptimal care	15
					Organ transplants	1
			Moral dimension	61	Organ transplants	30
					Euthanasia	25
			Emergency medicine regulations	31		
			Participant safety in clinical trials	70	Human medication debates	59
			Clinical trials in pharmacy	10	Participant safety	5
			Randomized clinical trials	5		
	Methodological innovation	615	Participatory research	122		
			Democratization of research	70		
			Internationalization of research	4		
			Empowerment	3		
			Cultural context in research	18		
			Ethical responsibility	397		
	Compensation ethics	15				
	Data security and privacy	31	Artificial intelligence	14		
			Big data	5		

## 4 Discussion

The discussion of the main findings will begin with a comment on the main descriptive statistics, the place of research ethics in some fields, the actual scientific debates, and the major research gaps.

### 4.1 What were the main bibliometric findings?

The overall presentation of the descriptive statistics of the database created for the bibliometric analysis shows that the notable citation count of 115,700 references in the dataset and the average rate of 17.36 citations per document highlight their significance within the academic community. This substantial citation rate indicates that the topic of research ethics is widely acknowledged and has considerable influence across a diverse range of subjects and disciplines (Tahamtan et al., 2016; Zhang et al., 2021). The greater number of automatically generated keywords (7,243) than author keywords (6,503) indicates that the algorithm employed by Biblioshiny to extract keywords offers a more comprehensive understanding of the document's content (Egozi et al., 2000). This shows the potential of these automatically generated keywords to provide a more informative representation of the research topics discussed in publications and the rich collaborative approach used.

Despite the wide variety of scientific fields that deal with ethical issues, medicine and human health research are mostly permeated by ethical considerations. These findings underscore the influence of ethical considerations in human health (Artal and Rubenfeld, 2017) research and across various fields because of the social context (Madushani, 2016). This emphasizes the importance of interdisciplinary collaboration in research to address ethical challenges (Laasch et al., 2023).

The results also show that the history of research ethics is a complex and evolving journey, marked by significant incidents and milestones in 1982, 1989, 1993, 1997, 2000, 2005, 2008, 2012, 2017, and 2020. These pivotal moments have shaped the intellectual landscape of research ethics and have profoundly impacted the global scientific community. As I delve into the intricate details of each of these incidents, it becomes evident that the evolution of research ethics is intertwined with the pursuit of social responsibility and justice (Dhai, 2014; IJsselmuiden et al., 2010). Over the last few decades, there has been increasing recognition of the necessity of ethics throughout the entire research process (Benatar and Singer, 2010). The ethical principles of respect include not only the research conducted, but also the data collection, analysis, and dissemination of research findings and innovation. Worldwide recognition of the scientific community has been driven by the expansion of research into various disciplines involving human participation (de Seneviratne, 2023). The Declaration of Helsinki has undergone seven revisions to ensure that it remains relevant and, to date, in the rapidly evolving field of medical research. Revisions were made to address criticism, incorporate new ethical considerations, and adapt to advancements in science and technology. These revisions also aimed to enhance the protection of research participants, particularly vulnerable groups, and address emerging issues, such as the use of biobanks and compensation for research-related injuries. The process of revising the Declaration involved input from expert stakeholders and organizations globally, as well as careful consideration of feedback and comments from various sources (Allebeck, 2009; Malik and Foster, 2016). Outside the field of medicine, the need to follow ethical research rules highlights gender and minority disparities in scientific publication bodies in medicine, pharmacy, and nursing (Bates et al., 2016; Dorrigan et al., 2022). Furthermore, the global landscape of research ethics has evolved significantly over the years, with an increasing emphasis on cross-cultural considerations and diverse ethical perspectives that shape research practices worldwide (Honan et al., 2013). As technology and scientific collaboration continue to transcend international boundaries, there is an evident need for a more comprehensive understanding of research ethics on a global scale (Adu-Gyamfi, 2015; Marshall and Batten, 2004). The period between 1965 and 2023 witnessed significant milestones in the evolution of research ethics, including the establishment of ethical review boards, the development of international guidelines such as the Declaration of Helsinki, and the integration of ethical considerations into research funding and publication policies. These developments have underscored the importance of ethical awareness and accountability in the scientific community, serving as a driving force for continued discourse and reflection on future research ethics.

The findings of the most relevant authors reveal that they made substantial contributions to their fields. This aligns with the notion that prolific authors often possess in-depth knowledge and experience of their respective domains (Boyack et al., 2005; Leydesdorff, 2011). This shows significant engagement in research ethics, reflecting a diverse range of perspectives and methodologies. This diversity is crucial for addressing the multifaceted nature of ethical challenges in science and underscores the collaborative and interdisciplinary nature of research ethics inquiry (Alfonso et al., 2019; Beshyah et al., 2018; Smith and Williams-Jones, 2012). This collaborative approach facilitates the exchange of ideas and fosters innovation in ethical theories and practices (Moreno-Cely et al., 2021). Overall, the prominence of these authors underscores the importance of their collective efforts to advance our understanding of research ethics and to promote ethical conduct across scientific disciplines.

The diversity of disciplinary fields is also apparent in the most relevant authors on the theme of research ethics. Their publications cover a wide variety of fields, despite the predominance of medicine. The observed transdisciplinary approach underscores the collaborative nature of addressing the ethical dilemmas in research (Mertz et al., 2014; Zhang et al., 2021). Notably, the absence of influential authors from agronomic science suggests a nascent exploration of ethical considerations within this discipline, signaling an opportunity for further investigation and integration of ethical frameworks (Bawden, 2010).

A total of 1,516 scientific publications hosted the documents collected in the database. The findings suggest that the frequency of journal publications by authors is influenced more by the disciplinary orientations and subjects covered by the journals than by the impact factors of those journals. This implies that authors are driven by the relevance of the journal to their research area rather than the prestige associated with high-impact factors (Mara et al., 2016). It underscores the importance of aligning research with the thematic focus of journals, reflecting the prioritization of content over journal metrics in scholarly communication (Bavdekar and Save, 2015). This observation highlights the nuanced factors that shape publication decisions among researchers across disciplines.

Research ethics have been the subject of research by several teams worldwide. The trend observed was that most publications were products of teams from the same country. The most notable countries in terms of publication volume were the United States, the United Kingdom, Canada, the Netherlands, Italy, Germany, South Africa, and China. South Saharan African countries were poorly represented in this study. This may be because of the ethic in research topic history regarding science progress and the secund World War, its atrocities, and the weak African implication. Countries that have experienced significant research ethics violations or scandals in the past tend to have more robust debates on this subject. It is the case in European Union countries with the atrocities of the Secund World War and the first formal declaration for physicians doing research, known as the Declaration of Helsinki. This is also the case in the United States and the history of controversial experiments, such as the Tuskegee Syphilis Study (Paul, 2018). Research ethics have evolved over time in response to various historical events and ethical dilemmas faced by researchers worldwide (Laudan, 1986).

For scientists such as Patelli et al. (2017), the level of scientific and technological advancement is the most relevant criterion for progress in science. For these authors, the global south is poorly represented in global scientific development, even if some countries from the BRICS group are present (Patelli et al., 2017). Science and its Critical Inquiry are philosophically different from many cultures worldwide. Some cultures emphasize individual rights, while others consider the social group highly. In the first case, research ethics can consider informed consent and privacy in research, and the other case must prioritize the collective benefits of science (Robert Nola, 2006; Tov and Nai, 2019; Xiao, 2021). Several other factors may have influenced the few publications on research ethics and the substantial number of publications, depending on the county. Political and economic factors, funding sources, weight of international collaboration, media attention, and the institutionalization of research ethics by the existence of ethics or bioethics committees and institutions.

The discussion of the results of the bibliometric analysis opens new perspectives for understanding the findings of the short systematic review. This highlights the current debates and gaps in the knowledge about research ethics.

### 4.2. Emergence of ethics and social responsibility as key themes in certain disciplines and new debates

Over the years, there has been a proliferation of research ethics publications that emphasize many debate topics among scientists.

One of the oldest but still topical issues in research ethics is informed consent and anonymity of research participants (Badampudi et al., 2022; Solis Sánchez et al., 2023). This author recalls the ethical imperatives of research stemming from the atrocities perpetrated by Nazi doctors regarding concentrating camp inmates. Prior to this publication, several authors addressed various aspects of this issue. These include Joffe and Miller (2008), who believes that medical research, although oriented toward care, fundamentally has a moral dimension distinct from the latter (Arahuete and Pinazo, 2022; Grosek et al., 2023). Some authors believes that the new regulations introduced in the United States since 1996 for emergency medicine to allow research on human subjects without their informed consent have shown great limitations because of the legal consequences of such research (Carracedo et al., 2024; Fritzsche, 2024; Orievulu et al., 2024).

Along the same lines Loveday et al. (2022) illustrated the case of research on adolescents without parental consent in South Africa (Loveday et al., 2022). The case of randomized clinical trials and the problem of suboptimal care (Zhang et al., 2023), that of consent given, and its limits in the case of organ transplants with living organ donors (Raza and Neuberger, 2022). Apart from debates in medicine and health sciences in general, discussions in the scientific world are also taking place in other disciplines. This is the case, for example, with the issue of methodological innovation in the social sciences, where questions on ethical responsibility, democratization of research, empowerment, and the relationship between innovation and the world of research are all on the agenda (Bellavista et al., 2022; Druckman and Donohue, 2020; Raza and Neuberger, 2022). A reference to the cases of action research highlights that, in the case of participatory research involving young people, the cultural context and moral values oblige the researcher to reach an agreement with his participants to facilitate their involvement in the research projects (Cullen and Walsh, 2020). For example, in psychology, the subject of protecting mentally incompetent patients has been addressed (Kaur, 2011). The remuneration or compensation of research subjects also raises ethical concerns because of the risk of transforming the payment received by participants into a simple commercial exchange (Różyńska, 2022). In the field of pharmacy, the question of participant safety during clinical trials is widely debated, especially for human medication (Jedličková, 2024). Apart from the many debates on Informed Consent and Participant Autonomy, data security and privacy are also emerging topics.

Khan et al. (2022) published one of the most important publications on this subject. He proposed to reflect on the ethics of research through the mechanisms of urbanization, such as smart cities that use artificial intelligence technologies. For this author, it was necessary to set security limits for the protection of personal data and the safety and security of these cities by forging effective political and algorithmic instruments (Bibri, 2021; Khan et al., 2022; Stahl and Eke, 2024). In the same vein, authors such as Calvo (2020) believe that the impacts and consequences of digital connectivity, the use of algorithms and databases in the urban digital society, and the security dangers of connected objects require rigorous application of ethical rules (Calvo, 2020).

Allam et al. (2022) and Bibri et al. (2022) believed that science and technology are the future of human living environments. They open an epistemological debate on the relationship between science and technology, and the profound changes they can exert on societies and their structures. They concluded that it is necessary to structure metaverses as virtual worlds in a manner that is morally acceptable and culturally appropriate for cities (Allam et al., 2022; Bibri, 2023; Bibri et al., 2022). Other authors have worked on the importance of the legal regulation of the Internet (Tzafestas, 2018) and ethical guidelines for data science training courses (Bates et al., 2020). Other topics of debate include the legal and social responsibility of digital companies (Lobschat et al., 2021) and ethics in communications, especially in print media (Kojo et al., 2022).

Artificial Intelligence and Machine Learning are also a major focus of scientific publications, in line with current debates on technology (Olteanu et al., 2019), ethical rules to be developed for machine learning and big data (Ananny and Crawford, 2018; Greene et al., 2019; Jobin and Vayena, 2019; Zwitter, 2014), and the use of artificial intelligence for predictions in politics, technology, and culture (Karppi, 2018; Morley et al., 2021; Lo Piano, 2020; Wong, 2020).

Review of ethics regulations and considerations of the ethics, accountability, and responsibility of Artificial Intelligence developers and users (Koniakou, 2023; Pant et al., 2022; Vesnic-Alujevic et al., 2020).

Research methodologies and ethical requirements have always contributed to the contributions of various authors. In this context, four major subthemes are widely discussed in the literature: (i) bias and fairness in research, (ii) access to research benefits, (iii) transparency and reproducibility, and (iv) ethics in global research collaboration. Over the past 5 years, publications dealing with bias and fairness in research results have focused on tools for measuring bias in various social sciences such as psychology, education sciences, and knowledge transfer in agriculture through digitalization (Fielke et al., 2020; Tay et al., 2022; Woo et al., 2022). Debates on bias and fairness are also taking place in computer sciences, machine learning, and artificial intelligence (Booth et al., 2021; Taylor, 2017), and their use in healthcare (Saheb et al., 2021).

In terms of access to and benefits from scientific research, early data protection throughout the entire research cycle is of paramount importance (Karcher et al., 2023). For other authors, the benefits of science and access cannot be achieved without promoting open science. However, the latter needs to be framed by ethical rules (Campbell et al., 2022, 2023). Financing the costs of publishing scientific articles and the implications for researchers' home countries can act as a brake on open access and reproducible research (Brabeck, 2021; Hardwicke et al., 2020; López-Nicolás et al., 2022; McKinley Yoder et al., 2022; Nwagwu, 2023; Page et al., 2021; Sabik et al., 2021; Sandoval-Lentisco et al., 2024). Discussions are also underway on issues related to gender and sexual violence in the academic environment (Roeschley et al., 2024; Siegel et al., 2021). Other authors make a plea for a global ethical governance of society capable of breaking down the barriers between bioethics, ecology, and society for a more effective theory of work (LeBlanc, 2023).

As concerns about the transparency and reproducibility of research have already been mentioned, I will quickly highlight a few salient points about ethics in global research collaborations. Over the past 5 years, several authors have discussed the importance of ethics in scientific research collaboration. Some believe that the criteria that determine collaboration between authors often vary according to their geographical and disciplinary proximity (Hedt-Gauthier et al., 2019). Similarly, some authors believe that collaborative research facilitates trust and scientific integrity while reducing ethical misconduct among researchers and research institutions (Bouter, 2022; Kerasidou, 2019, 2021; Kerasidou et al., 2021; Soehartono et al., 2022).

All of these topics of debate about ethics in science for better social responsibility and social justice are not exhaustive, but they shape major concerns. Nevertheless, it would be interesting to understand how social ethics and responsibility seem to be better positioned in the debates of some disciplines than others.

### 4.3 Knowledge gaps and future research pursuits

When I consider research ethics in science for better social responsibility and justice, there are several research gaps. This lack of scientific knowledge represents an area where further research is needed to ensure that science is ethically ruled to contribute to social wellbeing. Some of these gaps are as follows.

In many societies, there is no integration of traditional and scientific knowledge mechanisms (Kleiche-Dray et al., 2012). The knowledge produced by science and that generated by social institutions, traditions, habits, and customs of non-Western societies comes from different worlds and often does not combine easily. The aim of research ethics in promoting equitable access to scientific knowledge has not been sufficiently published. The awareness of the need to make scientific knowledge more accessible to all cultures of the world at all social levels, without segregation of any kind, including strategies for open access to scientific publications and inclusive representation in research (Broggiato et al., 2014; Chan et al., 2011; Kleiche-Dray et al., 2012; Kunz, 2021; Ross-Hellauer et al., 2022). Sub-Saharan African countries are particularly affected by this gap in the integration of science with local culture and language (Abolou, 2006). This gap must be addressed by researchers and research institutions. The need to take account of the particularities of different cultural groups in different parts of the world and to make scientific research culturally sensitive is also a gap in the current knowledge about research ethics, responsibility, and social justice (Kleiche-Dray et al., 2012; Robert Nola, 2006).

With great scientific progress in data management with big data and artificial intelligence in research, there is a gap in understanding and balancing data utilization with individual privacy rights and the rights of minorities and vulnerable populations (Lacroix, 2019; Nemati, 2020). By filling this gap, the global scientific community can provide richer answers to the research validation and methodological requirements. In this respect, there is a real need for more scientific publications on how to build research methodology, positive social sustainability, and ethics, beyond the simple production of research results (Rau et al., 2018).

The results obtained earlier in this study also show that there are gaps in these disciplines. In the Humanities and Social Sciences, despite the relevance of the issue of responsibility and social justice, the quantitative lack of publications (in this database of 3,128 publications, only 219 articles come from the field of Social Sciences) on research ethics is complemented by qualitative gaps. The latter can be seen in the growing need for publications on the ethical implications of qualitative research methods (Fielke et al., 2020; Tay et al., 2022; Woo et al., 2022), power dynamics in researcher- participant relationships (Kaaristo, 2022), and the recurring question of how to consider cultural sensitivity in cross-cultural studies (Marshall and Batten, 2004; Robert Nola, 2006).

In agricultural and food sciences, beyond the large group of natural and agricultural sciences, there are glaring gaps in research on ethics. The results showed that it was only in 2001 that the first scientific publication was published in this field. The article in question is titled ‘Ethics in apiculture by Wenning, Carl, published in the American Bee Journal. In this paper, the author discusses the code of conduct followed by the beekeepers. He defends the position that all professions should respect ethical rules and that agricultural professions should be no exception to this rule (Wenning, 2001).

The relative novelty of this theme in Agronomic Sciences makes it interesting to bridge the existing gaps in the ethical implications of the exercise of professionals (Wenning, 2001) linked to animal and fish production, plant production, natural resource conservation, and so on. The limits of knowledge can also be seen in the ethical implications of the development and dissemination of agricultural innovations (e.g., digitization of agriculture, GMOs, cloning of living material), the balance between food safety and preservation of the environment, and in the ethical considerations relating to animal-based agricultural research (Adalja et al., 2023; Hassoun et al., 2023; Häyry, 2018; Pollans, 2015; Rapela, 2020).

In view of the above, future research needs to focus on ethics, social responsibility, and social justice in the agricultural sciences, particularly in countries of the south, especially in sub-Saharan Africa.

### 4.4 Research limits

This contribution to the debate on research ethics attempts to take an overall look at the advances and gaps in scientific production on this topic.

It is important to acknowledge the limitations inherent in relying solely on the Scopus database for this mixed bibliometric analysis and systematic review. The observed growth in global research annual production and country contributions may be partially attributed to a “database effect” rather than solely reflecting actual increases in research output. Scopus has continuously expanded its journal coverage over time, which could artificially inflate growth trends.

Furthermore, it is possible to think that the Scopus database, because it contains a large volume of English-language publications, has an intrinsic bias toward this language and therefore, in turn, produces a bias toward publications from countries with an English tradition, such as Great Britain, the United States, Canada and Australia. This tendency may skew the representation of research output from non-English speaking countries and potentially underestimate their contributions to the field.

Furthermore, the exclusive use of Scopus may overlook relevant publications in journals not indexed by this database, potentially missing important contributions to research ethics. To mitigate these limitations, future studies should consider incorporating multiple databases, such as Web of Science, Dimensions or Google Scholar, to provide a more comprehensive and balanced view of the global research ethics landscape. Additionally, efforts to include non-English publications and analyze trends in publication language and country of origin over time would help contextualize the findings and provide a more accurate representation of the field's development.

## 5 Conclusion

Scientific research and its importance for human beings and societies oblige research professionals to take care of and apply ethics principles. This study set out to understand how ethical principles, scientists' social responsibility, and science's necessary social justice are internalized in many scientific fields worldwide. A focus was made about this topic analyzed across a database of 3,128 scientific publications from 1965 to 2023. Based on the outcomes, ethics and its principles influence research in human health and various other fields, owing to the social context and technological level. As I swim into the complex details of the main historical period of research ethics, it becomes evident that the evolution of research ethics is intertwined with the pursuit of social responsibility and justice. The main historical moment is: 1982, 1989, 1993, 1997, 2000, 2005, 2008, 2012, 2017, and 2020. During this period, significant developments were made in terms of research ethics. For example, there are the establishment of ethic committees, as the Declaration of Helsinki, and the integration of ethical considerations into research funding and publication practices These advancements have underscored the crucial importance of ethical awareness and accountability in the scientific domain, thereby stimulating ongoing discourse and self-reflection concerning research ethics. The proliferation of authors from various disciplines contributes to the complexity of the ethical challenges in science. Many authors prioritize journal relevance to research areas over impact factors when making publication decisions. The geographical map of research ethics publications shows that America leads research ethics publications, followed by Europe, Australia, Asia, and Africa. The current lack of understanding of ethical research practices, social responsibility, and the importance of cultural sensitivity in scientific research across various regions and cultural groups is a significant knowledge gap that needs to be addressed, particularly by South Saharan African researchers. Emerging topics such as data security, privacy, the ethical implications of AI, and digital connectivity animate scientific debate, even if there is insufficient knowledge production in agricultural science. However, this study is exploratory, mostly as a way to set an agenda for further research.

## Data Availability

The raw data supporting the conclusions of this article will be made available by the author, without undue reservation.

## References

[B1] Abolou C. R. (2006). L'Afrique, les langues et la société de la connaissance. Hermes Revue 45, 165–172. 10.4267/2042/24047

[B2] Adalja A. Liaukonyte J. Wang E. Zhu X. (2023). GMO and Non-GMO labeling effects: evidence from a quasi-natural experiment. Market. Sci. 42:1375. 10.1287/mksc.2022.137519642375

[B3] Adu-Gyamfi J. (2015). Ethical challenges in cross-cultural field research: a comparative study of UK and Ghana. Available at: https://www.researchgate.net/publication/277586639 (accessed August 10, 2024).

[B4] Afolabi M. O. (2012). Researching the vulnerables: issues of consent and ethical approval. Afr. J. Med. Med. Sci. 41. 23678631

[B5] Agrahari A. K. Sharma H. K. (2017). Plagiarism and its aspects in world academic community: a review. Pharma Times 49.

[B6] Alfonso F. Zelveian P. Monsuez J. J. Aschermann M. Boehm M. Hernandez A. B. . (2019). Authorship: from credit to accountability reflections from the editors' network. Anatol. J. Cardiol. 21, 281–286. 10.1007/s12471-019-1273-y 31062751 PMC6528517

[B7] Allam Z. Sharifi A. Bibri S. E. Jones D. S. Krogstie J. (2022). The metaverse as a virtual form of smart cities: opportunities and challenges for environmental, economic, and social sustainability in urban futures. Smart Cities. 2:22. 10.3390/smartcities5030040

[B8] Allebeck P. (2009). A new Helsinki Declarationbut what about public health research. Eur. J. Public Health 19:129. 10.1093/eurpub/ckp036 19307246

[B9] Ambroz M. Bukovec B. (2015). Fears of a modern society. Innov. Issues Approach. Soc. Sci. 8, 163–179. 10.12959/issn.1855-0541.IIASS-2015-no1-art10

[B10] Amutuhaire T. (2022). The reality of the ‘publish or perish’ concept, perspectives from the global south. Publish. Res. Q. 38. 10.1007/s12109-022-09879-0

[B11] Ananny M. Crawford K. (2018). *Seeing without* knowing: Limitations of the transparency ideal and its application to algorithmic accountability. New Media Soc. 20, 973–989. 10.1177/1461444816676645

[B12] Arahuete L. Pinazo D. (2022). The effect of mindfulness training on the self-regulation of socio-moral thoughts. Psychol. Rep. 127, 2898–2917. 10.31124/advance.20321709.v1 36573303

[B13] Armond A. C. V. Gordijn B. Lewis J. Hosseini M. Bodnár J. K. Holm S. . (2021). A scoping review of the literature featuring research ethics and research integrity cases. BMC Med. Ethics 22, 1–14. 10.1186/s12910-021-00620-8 33931043 PMC8086087

[B14] Artal R. Rubenfeld S. (2017). Ethical issues in research. Best Pract. Res. Clin. Obstet. Gynaecol. 43, 107–114. 10.1016/j.bpobgyn.2016.12.006 28190696

[B15] Ataullahjan A. Lo S. Aziz M. H. Somani N. A. Bhutta Z. A. (2022). Why we need stricter oversight of research involving human subjects affected by conflict. AMA J. Ethics 24, E518–E529. 10.1001/amajethics.2022.518 35713919

[B16] Badampudi D. Fotrousi F. Cartaxo B. Usman M. (2022). Reporting consent, anonymity and confidentiality procedures adopted in empirical studies using human participants. e-Informatica Softw. Eng. J. 16. 10.37190/e-Inf220109

[B17] Bain L. E. Ebuenyi I. D. Ekukwe N. C. Awah P. K. (2018). Rethinking research ethics committees in low-and medium-income countries. Res. Ethics 14:174701611769202. 10.1177/1747016117692026

[B18] Bates C. Gordon L. Travis E. Chatterjee A. Chaudron L. Fivush B. . (2016). Striving for gender equity in academic medicine careers: a call to action. Acad. Med. 91, 1050–1052. 10.1097/ACM.0000000000001283 27332868 PMC5954825

[B19] Bates J. Cameron D. Checco A. Clough P. D. Hopfgartner F. Mazumdar S. . (2020). “Integrating FATE/critical data studies into data science curricula: where are we going and how do we get there?” in FAT^*^ (New York, NY: ACM). 10.1145/3351095.3372832

[B20] Bavdekar S. Save S. (2015). Choosing the right journal for a scientific paper. J. Assoc. Phys. India 63 26710401

[B21] Bawden R. (2010). Ethics and agricultural science: a worldview challenge. Agric. Sci. 22, 19–22. 10.3316/informit.609634535239808

[B22] Becker A. Lukka K. (2022). Instrumentalism and the publish-or-perish regime. Crit. Perspect. Account. 94:102436. 10.1016/j.cpa.2022.102436

[B23] Bellavista J. Elboj-Saso C. García Yeste C. Villarejo-Carballido B. (2022). Innovative methodological approach to analyze innovation and social impact. Int. J. Qual. Methods 21. 10.1177/16094069221083373

[B24] Benatar S. R. Singer P. A. (2010). Responsibilities in international research: a new look revisited. J. Med. Ethics 36, 194–197. 10.1136/jme.2009.032672 20338927

[B25] Berger-Tal O. Greggor A. L. Macura B. Adams C. A. Blumenthal A. Bouskila A. . (2019). Systematic reviews and maps as tools for applying behavioral ecology to management and policy. Behav. Ecol. 30, 1–8. 10.1093/beheco/ary130

[B26] Beshyah S. Ibrahim W. Aburawi E. Elkhammas E. (2018). The rules and realities of authorship in biomedical journals: a cautionary tale for aspiring researchers. Ibnosina J. Med. Biomed. Sci. 10, 149–157. 10.4103/ijmbs.ijmbs_62_18

[B27] Bibri S. E. (2021). *Data*-driven smart eco-cities and sustainable integrated districts: a best-evidence synthesis approach to an extensive literature review. Eur. J. Futures Res. 9:16. 10.1186/s40309-021-00181-4

[B28] Bibri S. E. (2023). The metaverse as a virtual model of platform urbanism: its converging AIoT, XReality, neurotech, and nanobiotech and their applications, challenges, and risks. Smart Cities. 6, 1345–1384 10.3390/smartcities6030065

[B29] Bibri S. E. Allam Z. Bibri S. E. Allam Z. (2022). The Metaverse as a virtual form of data-driven smart cities: the ethics of the hyper-connectivity, datafication, algorithmization, and platformization of urban society. Comput. Urban Sci. 2:22. 10.1007/s43762-022-00050-1 35915731 PMC9330959

[B30] Bonde S. Briant C. Firenze P. Hanavan J. Huang A. Li M. . (2016). Making choices: ethical decisions in a global context. Sci. Eng. Ethics 22, 343–366. 10.1007/s11948-015-9641-5 25962719

[B31] Booth B. M. Hickman L. Subburaj S. K. Tay L. Woo S. E. D'Mello S. . (2021). “Bias and fairness in multimodal machine learning: a case study of automated video interviews,” in International Conference on Multimodal Interaction (Montréal, QC). 10.1145/3462244.3479897

[B32] Boulesteix A. L. Hoffmann S. Charlton A. Seibold H. (2020). A replication crisis in methodological research? Significance 17:1444. 10.1111/1740-9713.01444

[B33] Bouter L. (2022). “What research institutions can do to Foster research integrity,” in The 92nd Dies Natalis of Tilburg University, Tilburg, The Netherlands, 10.1007/978-3-030-99680-2_5931965429

[B34] Boyack K. W. Klavans R. Börner K. (2005). Mapping the backbone of science. Scientometrics 64, 351–374. 10.1007/s11192-005-0255-6

[B35] Brabeck M. (2021). Open science and feminist ethics: promises and challenges of open access. Psychol. Women Q. 45:457. 10.1177/03616843211030926

[B36] Broggiato A. Arnaud-Haond S. Chiarolla C. Greiber T. (2014). Fair and equitable sharing of benefits from the utilization of marine genetic resources in areas beyond national jurisdiction: bridging the gaps between science and policy. Mar. Policy 49, 176–185. 10.1016/j.marpol.2014.02.012

[B37] Calvo P. (2020). The ethics of Smart City (EoSC): moral implications of hyperconnectivity, algorithmization and the datafication of urban digital society. Ethics Inf. Technol. 22. 10.1007/s10676-019-09523-0

[B38] Campbell R. Goodman-Williams R. Javorka M. Engleton J. Gregory K. (2022). Understanding sexual assault survivors' perspectives on archiving qualitative data: implications for feminist approaches to open science. Psychol. Women Q. 47, 51–64. 10.1177/03616843221131546

[B39] Campbell R. Javorka M. Engleton J. Fishwick K. Gregory K. Goodman-Williams R. . (2023). Open-science guidance for qualitative research: an empirically validated approach for de-identifying sensitive narrative data. Adv. Methods Pract. Psychol. Sci. 6:25152459231205832. 10.1177/25152459231205832

[B40] Cantley I. (2023). Replicable quantitative psychological and educational research: Possibility or pipe dream? Educ. Philos. Theory 55:2090926. 10.1080/00131857.2022.2090926

[B41] Carracedo S. Palmero A. Saenz C. (2024). “Ethics review challenges,” in Research Ethics in Epidemics and Pandemics: A Casebook. Public Health Ethics Analysis, eds. S. Bull, M. Parker, J. Ali, M. Jonas, V. Muthuswamy, C. Saenz, et al. (Cham: Springer), 107–130. 10.1007/978-3-031-41804-4_6

[B42] Chan L. Kirsop B. Arunachalam S. (2011). Toward open and equitable access to research and knowledge for development. PLoS Med. 8:e1001016. 10.1371/journal.pmed.1001016 21483470 PMC3066136

[B43] Considine J. Shaban R. Z. Fry M. Curtis K. (2017). Evidence based emergency nursing: designing a research question and searching the literature. Int. Emerg. Nurs. 32, 78–82. 10.1016/j.ienj.2017.02.001 28233626

[B44] Cooper M. M. (2018). The replication crisis and chemistry education research. J. Chem. Educ. 95, 1–2. 10.1021/acs.jchemed.7b00907

[B45] Cullen O. Walsh C. A. (2020). A narrative review of ethical issues in participatory research with young people. Young 28, 363–386. 10.1177/1103308819886470

[B46] Dal-Ré R. Bouter L. M. Cuijpers P. Gluud C. Holm S. (2020). Should research misconduct be criminalized? Res. Ethics 16:174701611989840. 10.1177/1747016119898400

[B47] De Kanter A. F. J. Jongsma K. R. Verhaar M. C. Bredenoord A. L. (2023). The ethical implications of tissue engineering for regenerative purposes: a systematic review. Tissue Eng. B: Rev. 29:33. 10.1089/ten.teb.2022.0033 36112697 PMC10122262

[B48] de Seneviratne R. A. (2023). Ethics of research. J. Natl. Sci. Found. Sri Lanka 51, 183–184. 10.4038/jnsfsr.v51i2.11747

[B49] de Zárate Alcarazo L. O. (2022). Explainable artificiai intelligence. Eunomia. Rev. Cult. Legalidad 22.

[B50] Dhai A. (2014). The research ethics evolution: from Nuremberg to Helsinki. S. Afr. Med. J. 104:7864. 10.7196/SAMJ.7864 24897818

[B51] Dorrigan A. Zuccala E. Talley N. J. (2022). Striving for gender equity at the Medical Journal of Australia. Med. J. Aust. 217, 138–139. 10.5694/mja2.51642 35908263

[B52] Druckman D. Donohue W. (2020). Innovations in social science methodologies: an overview. Am. Behav. Sci. 64, 3–18. 10.1177/0002764219859623

[B53] Edwards M. R. Charlwood A. Guenole N. Marler J. (2022). HR analytics: an emerging field finding its place in the world alongside simmering ethical challenges. Hum. Resour. Manag. J. 34. 10.1111/1748-8583.12435

[B54] Egozi O. Markovitch S. Gabrilovich E. (2000). Concept-based ir using explicit semantic analysis. ACM Trans. Inf. Syst. 29, 1–34.

[B55] Fairhall A. Svoboda K. Nobre A. C. (2016). Global collaboration, learning from other fields. Neuron 92, 561–563. 10.1016/j.neuron.2016.10.040 27809993

[B56] Fielke S. Taylor B. Jakku E. (2020). Digitalisation of agricultural knowledge and advice networks: a state-of-the-art review. Agric. Syst. 180:102763. 10.1016/j.agsy.2019.102763

[B57] Franzke A. S. (2022). An exploratory qualitative analysis of AI ethics guidelines. J. Inf. Commun. Ethics Soc. 20. 10.1108/JICES-12-2020-0125

[B58] Fritzsche L. (2024). Research in times of emergency: methodological and ethical considerations in immigrant and refugee research. Geogr. Rev. 114, 1–18. 10.1080/00167428.2024.2329598

[B59] Ghose T. Shubert V. Chaudhuri S. Poitevien V. Updyke A. (2021). Are financial incentives appropriate means of encouraging medication adherence among people living with HIV? AMA J. Ethics 23:394. 10.1001/amajethics.2021.394 34038347

[B60] Girgin U. Acar Y. Akbaş E. Yavuz E. Altan A. E. Boran M. . (2022). Conversation analysis methodology: validity, reliability, and ethical issues in data collection and analysis procedures. Hacettepe Egitim Dergisi, 37.

[B61] Golden J. Mazzotta C. M. Zittel-Barr K. (2023). Systemic obstacles to addressing research misconduct in higher education: a case study. J. Acad. Ethics 21, 71–82. 10.1007/s10805-021-09438-w 34483786 PMC8403249

[B62] Goldenberg M. J. (2022). Public trust in science. Interdiscip. Sci. Rev. 48, 1–13. 10.1080/03080188.2022.2152243

[B63] Greene D. Hoffmann A. Stark L. (2019). Better, Nicer, Clearer, Fairer: A Critical Assessment of the Movement for Ethical Artificial Intelligence and Machine Learning (Grand Wailea, HI: HICSS). 10.24251/HICSS.2019.258

[B64] Grosek S. Pleterski Rigler D. Podbregar M. Erčulj V. (2023). Knowledge of and attitudes toward medical research ethics among first year doctoral students in Slovenia at the Faculty of Medicine, University of Ljubljana. BMC Med. Educ. 23:828. 10.1186/s12909-023-04809-w 37924025 PMC10623751

[B65] Hardwicke T. E. Wallach J. D. Kidwell M. C. Bendixen T. Crüwell S. Ioannidis J. P. A. . (2020). An empirical assessment of transparency and reproducibility-related research practices in the social sciences (2014-2017). R. Soc. Open Sci. 7:190806. 10.1098/rsos.190806 32257301 PMC7062098

[B66] Hassan R. Zain F. M. Bakar K. A. Kamaruzaman A. F. Jamsari E. A. Lailatul N. . (2022). The challenges in the development of ethics and moral values among technical and vocational education and training (TVET) undergraduate students. J. Posit. Scho. Psychol. 2022.

[B67] Hassoun A. Marvin H. J. P. Bouzembrak Y. Barba F. J. Castagnini J. M. Pallarés N. . (2023). Digital transformation in the agri-food industry: recent applications and the role of the COVID-19 pandemic. Front. Sustain. Food Syst. 7:1217813. 10.3389/fsufs.2023.1217813

[B68] Haven T. van Woudenberg R. (2021). Explanations of research misconduct, and how they hang together. J. Gen. Philos. Sci. 52, 543–561. 10.1007/s10838-021-09555-5

[B69] Hawkins M. Elsworth G. R. Osborne R. H. (2018). Application of validity theory and methodology to patient-reported outcome measures (PROMs): building an argument for validity. Qual. Life Res. 27, 1695–1710. 10.1007/s11136-018-1815-6 29464456 PMC5997725

[B70] Häyry M. (2018). Ethics and cloning. Br. Med. Bull. 128, 15–21. 10.1093/bmb/ldy031 30203088

[B71] Hedt-Gauthier B. Jeufack H. Neufeld N. Alem A. Sauer S. Odhiambo J. . (2019). Stuck in the middle: a systematic review of authorship in collaborative health research in Africa, 2014–2016. BMJ Glob. Health 4:e001853. 10.1136/bmjgh-2019-001853 31750000 PMC6830050

[B72] Honan E. Hamid M. O. Alhamdan B. Phommalangsy P. Lingard B. (2013). Ethical issues in cross-cultural research. Int. J. Res. Method Educ. 36, 386–399. 10.1080/1743727X.2012.705275

[B73] Hope D. Dewar A. Hay C. (2021). Is there a replication crisis in medical education research? Acad. Med. 96, 958–963. 10.1097/ACM.0000000000004063 33735127

[B74] Horbach S. P. J. M. Breit E. Halffman W. Mamelund S. E. (2020). On the willingness to report and the consequences of reporting research misconduct: the role of power relations. Sci. Eng. Ethics 26, 1595–1623. 10.1007/s11948-020-00202-8 32103454 PMC7286863

[B75] Huber B. Barnidge M. de Zúñiga H. G. Liu J. (2019). Fostering public trust in science: the role of social media. Public Underst. Sci. 28, 759–777. 10.1177/0963662519869097 31524092

[B76] IJsselmuiden C. B. Kass N. E. Sewankambo K. N. Lavery J. V. (2010). Evolving values in ethics and global health research. Glob. Public Health 5, 154–163. 10.1080/17441690903436599 20213565

[B77] Intemann K. (2023). Science communication and public trust in science. Interdiscip. Sci. Rev. 48, 1–16. 10.1080/03080188.2022.2152244

[B78] Jay M. Orstad S. L. Wali S. Wylie-Rosett J. Tseng C. H. Sweat V. . (2019). Goal-directed vs. outcome-based financial incentives for weight loss among low-income patients with obesity: Rationale and design of the Financial Incentives for Weight Reduction (FIReWoRk) randomised controlled trial. BMJ Open 9:e025278. 10.1136/bmjopen-2018-025278 30962231 PMC6500238

[B79] Jedličková A. (2024). Ethical aspects of conducting clinical trials of human medicinal products. Ceska Slov Farm. 72, 256–266. 38346903

[B80] Jobin A. Vayena E. (2019). The global landscape of AI ethics guidelines. https://www.nature.com/natmachintell Nat. Mach. Intell. 1, 389–399. 10.1038/s42256-019-0088-2

[B81] Joffe S. Miller F. (2008). Bench to Bedside: Mapping the Moral Terrain of Clinical Research. The Hastings Center Report. 10.1353/HCR.2008.0019 18457227

[B82] Kaaristo M. (2022). Everyday power dynamics and hierarchies in qualitative research: the role of humour in the field. Qual. Res. 22, 743–760. 10.1177/14687941221096597

[B83] Karcher S. Secen S. Weber N. (2023). Protecting sensitive data early in the research data lifecycle. J. Priv. Confid. 13. 10.29012/jpc.846

[B84] Karppi T. (2018). “The computer said so”: on the ethics, effectiveness, and cultural techniques of predictive policing. Soc. Media Soc. 4:205630511876829. 10.1177/2056305118768296

[B85] Kaur S. (2011). The adequacy of the ethics review process in Malaysia : protection of the interests of mentally incapacitated adults who enrol in clinical trials. London: University College London.

[B86] Kerasidou A. (2019). The role of trust in global health research collaborations. Bioethics 33, 495–501. 10.1111/bioe.12536 30480821 PMC6563149

[B87] Kerasidou A. (2021). “Trustworthy institutions in global health research collaborations,” in The Cambridge Handbook of Health Research Regulation, eds. G. Laurie, E. Dove, A. Ganguli-Mitra, C. McMillan, E. Postan, N. Sethi, and A. Sorbie (Cambridge: Cambridge University Press). 10.1017/9781108620024.011

[B88] Kerasidou C. Kerasidou A. Buscher M. Wilkinson S. (2021). Before and beyond trust: reliance in medical AI. J. Med. Ethics 48, 852–856. 10.1136/medethics-2020-107095 34426519 PMC9626908

[B89] Khan K. Maabreh M. Ghaly M. Khan K. Qadir J. Al-Fuqaha A. . (2022). Developing future human-centered smart cities: Critical analysis of smart city security, Data management, and Ethical challenges.

[B90] Kleiche-Dray M. Waast R. Katz E. Georges I. Lazos E. Arrellano A. . (2012). Integrating traditional and scientific knowledge(s) for an equitable and sustainable use of natural resources: analytical framework report. IRD.

[B91] Kojo M. Vilhunen T. Kari M. Litmanen T. Lehtonen M. Kojo M. . (2022). The art of being ethical and responsible: print media debate on final disposal of spent nuclear fuel in Finland and Sweden. Soc. Just. Res. 35, 157–187. 10.1007/s11211-022-00391-6

[B92] Koniakou V. (2023). From the “rush to ethics” to the “race for governance” in Artificial Intelligence. New York, NY. 10.1007/s10796-022-10300-6

[B93] Kunz R. (2021). Opening access, closing the knowledge Gap? Analysing GC No. 25 on the right to science and its implications for the global science system in the digital age. Z. Ausländisch. 81, 23–46. 10.17104/0044-2348-2021-1-23

[B94] Kwame A. Petrucka P. M. (2023). Ethical dilemmas in cross-national qualitative research: a reflection on personal experiences of ethics from a doctoral research project. J. Acad. Ethics. 22, 251–268. 10.1007/s10805-023-09484-6

[B95] Laasch O. Moosmayer D. C. Antonacopoulou E. P. (2023). The interdisciplinary responsible management competence framework: an integrative review of ethics, responsibility, and sustainability competences. J. Bus. Ethics 187, 733–757. 10.1007/s10551-022-05261-436521423

[B96] Lacroix P. (2019). “Big data privacy and ethical challenges,” in Lecture Notes in Bioengineering (Cham: Springer), 101–111. 10.1007/978-3-030-06109-8_9

[B97] Langlais P. J. (2006). Ethics for the next generation. Chron. High. Educ. 52.

[B98] Larsson S. (2020). On the governance of artificial intelligence through ethics guidelines. Asian J. Law Soc. 7:19. 10.1017/als.2020.19

[B99] Laskar M. S. (2017). Publishing articles in scientific journals: a concern for research misconduct or dishonesty (fabrication, falsification and plagiarism). Mediscope 4:34995. 10.3329/mediscope.v4i2.34995

[B100] Lau P. (2021). A case study on research postgraduate students' understanding of academic integrity at a Hong Kong University. Front. Educ. 6:647626. 10.3389/feduc.2021.647626

[B101] Laudan L. (1986). Science and Values. The Aims of Science and Their Role in Scientific Debate. Oakland, CA: University of California Press. 10.1525/9780520908116

[B102] Laws A. L. S. Utne T. (2019). Ethics guidelines for immersive journalism. Front. Robot. AI 6:28. 10.3389/frobt.2019.00028 33501044 PMC7805689

[B103] LeBlanc A. B. (2023). At the confluence of ethics, laws and society: global working theory merging bio-ethics. SN Soc. Sci. 4:5.

[B104] Leydesdorff L. (2011). Katy Börner: atlas of science: visualizing what we know: the MIT Press, Cambridge, MA/London, UK, 2010, US$20. Scientometrics 88, 675–677. 10.1007/s11192-011-0409-7 21836764 PMC3125506

[B105] Lin L. Chu H. (2018). Quantifying publication bias in meta-analysis. Biometrics 74:12817. 10.1111/biom.12817 29141096 PMC5953768

[B106] Lo Piano S. (2020). Ethical principles in machine learning and artificial intelligence: cases from the field and possible ways forward. Humanit. Soc. Sci. Commun. 7:9. 10.1057/s41599-020-0501-9

[B107] Lobschat L. Mueller B. Eggers F. Brandimarte L. Diefenbach S. Kroschke M. . (2021). Corporate digital responsibility. J. Bus. Res. 122, 875–888. 10.1016/j.jbusres.2019.10.006

[B108] López-Nicolás R. López-López J. Rubio-Aparicio M. Sánchez-Meca J. (2022). A meta-review of transparency and reproducibility-related reporting practices in published meta-analyses on clinical psychological interventions (2000–2020). Behav. Res. Methods. 54, 334–339. 10.31234/osf.io/dz8cm 34173943 PMC8863703

[B109] Loveday M. Goga A. Dhai A. Labuschaigne M. Roussouw T. Burgess T. . (2022). Ethically acceptable consent approaches to adolescent research in South Africa. South. Afr. J. HIV Med. 23:1385. 10.4102/sajhivmed.v23i1.1385 36299555 PMC9558167

[B110] Madushani H. D. P. (2016). Ethical issues in social science research: a review. J. Soc. Stat. 3.

[B111] Malik A. Y. Foster C. (2016). The revised Declaration of Helsinki: cosmetic or real change? J. R. Soc. Med. 109, 184–189. 10.1177/0141076816643332 27150712 PMC4872207

[B112] Malviya A. (2012). Peer review in scientific publications. Bone Joint 360:1. 10.1302/2048-0105.11.360017

[B113] Mara L. Figueiredo A. Luiza A. Palma R. Fernandes Araujo C. De Fátima J. . (2016). Aspects affecting the choice for scientific journal publishing. Braz. Dent. Sci. 19:1238. 10.14295/bds.2016.v19i2.1238

[B114] Marshall A. Batten S. (2004). Researching Across Cultures: Issues of Ethics and Power. Available at: http://www.qualitative-research.net/fqs/ (accessed April 22, 2024).

[B115] Martínez-Valdivia E. Pegalajar-Palomino M. C. Burgos-García A. (2020). Social responsibility and university teacher training: keys to commitment and social justice into schools. Sustainability 12, 1–15. 10.3390/su1215617935136666

[B116] Matosas-López L. Cuevas-Molano E. (2022). Assessing teaching effectiveness in blended learning methodologies: validity and reliability of an instrument with behavioral anchored rating scales. Behav. Sci. 12:394. 10.3390/bs12100394 36285963 PMC9598132

[B117] McKinley Yoder C. Soule I. Nguyen C. Saluta I. (2022). Ethical global health in nursing education: an integrative review. Nurse Educ. Pract. 58:103263. 10.1016/j.nepr.2021.103263 34891027

[B118] Mertz M. Inthorn J. Renz G. Rothenberger L. G. Salloch S. Schildmann J. . (2014). Research across the disciplines: a road map for quality criteria in empirical ethics research. BMC Med. Ethics 15:17. 10.1186/1472-6939-15-17 24580847 PMC3974020

[B119] Mills T. Massoumi N. Miller D. (2020). The ethics of researching ‘terrorism’ and political violence: a sociological approach. Contemp. Soc. Sci. 15, 119–133. 10.1080/21582041.2019.1660399

[B120] Mohammed Z. Abdelsalam E. M. (2022). Research misconduct in developing countries. Egypt. J. Intens. Care Emerg. Med. 2:1000. 10.21608/jicem.2022.136048.1000

[B121] Moore L. Savage J. (2002). Participant observation, informed consent and ethical approval. Nurse Res. 9, 58–69. 10.7748/nr2002.07.9.4.58.c6198 12149897

[B122] Moreno-Cely A. Cuajera-Nahui D. Escobar-Vasquez C. G. Vanwing T. Tapia-Ponce N. (2021). Breaking monologues in collaborative research: bridging knowledge systems through a listening-based dialogue of wisdom approach. Sustain. Sci. 16, 919–931. 10.1007/s11625-021-00937-8

[B123] Morley J. Cowls J. Floridi L. Taddeo M. Tsamados A. Roberts H. . (2021). The ethics of algorithms: key problems and solutions. Philos. Stud.vol paf Ser.

[B124] Nemati H. (2020). Pervasive Information Security and Privacy Developments: Trends and Advancements. New York, NY: Quantum.

[B125] Nwagwu W. (2023). Nature and characteristics of global attention to research on article processing charges. J. Acad. Librariansh. 49:102808. 10.1016/j.acalib.2023.102808

[B126] Nyström M. E. Karltun J. Keller C. Gäre B. A. (2018). Collaborative and partnership research for improvement of health and social services: researcher's experiences from 20 projects. Health Res. Policy Syst. 16:46. 10.1186/s12961-018-0322-0 29843735 PMC5975592

[B127] Olteanu A. Castillo C. Diaz F. Kiciman E. (2019). Social data: biases, methodological pitfalls, and ethical boundaries. Front. Big Data. 2:13. 10.3389/fdata.2019.00013 33693336 PMC7931947

[B128] Orievulu K. Hinga A. Nkosi B. Ngwenya N. Seeley J. Akanlu A. . (2024). A scoping review of ethics review processes during public health emergencies in Africa. BMC Med. Ethics 25. 10.1186/s12910-024-01054-8 38778293 PMC11110293

[B129] Page M. J. Moher D. Fidler F. Higgins J. P. T. Brennan S. E. Haddaway N. R. . (2021). The REPRISE project: protocol for an evaluation of reproducibility and replicability in syntheses of evidence. Syst. Rev. 10:112. 10.1186/s13643-021-01670-0 33863381 PMC8052676

[B130] Pant A. Hoda R. Tantithamthavorn C. Turhan B. (2022). Ethics in AI through the developer's prism: a socio-technical grounded theory literature review and guidelines. arXiv. [Preprint]. 10.48550/2206.09514

[B131] Patelli A. Cimini G. Pugliese E. Gabrielli A. (2017). The scientific influence of nations on global scientific and technological development. J. Informetr. 11, 1229–1237. 10.1016/j.joi.2017.10.005

[B132] Paul H. (2018). The scientific self: reclaiming its place in the history of research ethics. Sci. Eng. Ethics 24, 1379–1392. 10.1007/s11948-017-9945-8 28721643 PMC6209027

[B133] Pollans M. J. (2015). Regulating farming: balancing food safety and environmental protection in a cooperative governance regime. Wake Forest Legal Rev. 50.

[B134] Pollock A. Berge E. (2018). How to do a systematic review. Int. J. Stroke 13, 138–156. 10.1177/1747493017743796 29148960

[B135] Quester P. G. Simpson J. (1998). International marketing ethics: a cross-cultural study. Austral. Mark. J. 6, 51–61. 10.1016/S1441-3582(98)70249-6

[B136] Rapela M. A. (2020). Fostering Innovation for Agriculture 4.0: A Comprehensive Plant Germplasm System. New York, NY. 10.1007/978-3-030-32493-3

[B137] Rau H. Goggins G. Fahy F. (2018). From invisibility to impact: recognising the scientific and societal relevance of interdisciplinary sustainability research. Res. Policy 47, 266–276. 10.1016/j.respol.2017.11.005

[B138] Rawwas M. Y. A. Swaidan Z. Oyman M. (2005). Consumer ethics: a cross-cultural study of the ethical beliefs of Turkish and American consumers. J. Bus. Ethics 57, 183–195. 10.1007/s10551-004-5092-7

[B139] Raza F. Neuberger J. (2022). Consent in organ transplantation: putting legal obligations and guidelines into practice. BMC Med. Ethics 23, 1–10. 10.1186/s12910-022-00791-y 35790956 PMC9255499

[B140] Reisig M. D. Holtfreter K. Berzofsky M. E. (2020). Assessing the perceived prevalence of research fraud among faculty at research-intensive universities in the USA. Account. Res. 27:1772060. 10.1080/08989621.2020.1772060 32438829

[B141] Resnik D. (2023). Disclosing and managing non-financial conflicts of interest in scientific publications. Res. Ethics 19. 10.1177/17470161221148387 37621567 PMC10448996

[B142] Różyńska J. (2022). The ethical anatomy of payment for research participants. Med. Health Care Philos. 25, 449–464 10.1007/s11019-022-10092-1 35610403 PMC9427899

[B143] Robert Nola G. I. (2006). Philosophy, Science, Education and Culture. Cham: Springer.

[B144] Rodrigues F. Gupta P. Khan A. P. Chatterjee T. Sandhu N. K. Gupta L. . (2023). The cultural context of plagiarism and research misconduct in the Asian region. J. Korean Med. Sci. 38:e88. 10.3346/jkms.2023.38.e88 36974397 PMC10042729

[B145] Roeschley A. Miller J. Nikitopoulos A. Gieringer M. Holden J. (2024). *Archiving difficult* realities: a systematic investigation of records related to sexual violence in US college and university archives. Arch. Sci. 24, 387–414. 10.1007/s10502-024-09434-0

[B146] Ross-Hellauer T. Reichmann S. Cole N. L. Fessl A. Klebel T. Pontika N. . (2022). Dynamics of cumulative advantage and threats to equity in open science: a scoping review. R. Soc. Open Sci. 9:211032. 10.1098/rsos.211032 35116143 PMC8767192

[B147] Ryan J. C. Tipu S. A. A. (2022). Business and management research: low instances of replication studies and a lack of author independence in replications. Res. Policy 51:104408. 10.1016/j.respol.2021.104408

[B148] Sabik N. Matsick J. L. McCormick-Huhn K. Cole E. (2021). Bringing an intersectional lens to “open” science: an analysis of representation in the reproducibility project. Psychol. Women Q. 45, 475–492. 10.1177/03616843211035678

[B149] Saheb T. Saheb T. Carpenter D. O. (2021). Mapping research strands of ethics of artificial intelligence in healthcare: a bibliometric and content analysis. Comput. Biol. Med. 135:104660. 10.1016/j.compbiomed.2021.104660 34346319

[B150] Sandoval-Lentisco A. López-Nicolás R. Tortajada M. López-López J. A. Meca J. S. (2024). Transparency in cognitive training meta-analyses: a meta-review. Neuropsychol. Rev. 10.1007/s11065-024-09638-2 38639881 PMC12328470

[B151] Santos J. Palumbo F. Molsen-David E. Willke R. J. Binder L. Drummond M. . (2017). ISPOR Code of Ethics 2017 (4th Edition). Value Health 20, 1227–1242. 10.1016/j.jval.2017.10.018 29241881

[B152] Seifert S. G. LaMothe E. G. Schmitt D. B. (2023). Perceptions of the ethical infrastructure, professional autonomy, and ethical judgments in accounting work environments. J. Bus. Ethics 182. 10.1007/s10551-021-05001-0

[B153] Shaw D. (2019). The quest for clarity in research integrity: a conceptual schema. Sci. Eng. Ethics 25, 1085–1093. 10.1007/s11948-018-0052-2 29594670

[B154] Siegel J. A. Calogero R. M. Eaton A. Roberts T. (2021). Identifying gaps and building bridges between feminist psychology and open science. Psychol. Women Q. 45:036168432110444. 10.1177/03616843211044494

[B155] Sivasubramaniam S. D. Cosentino M. Ribeiro L. Marino F. (2021). Unethical practices within medical research and publication – an exploratory study. Int. J. Educ. Integr. 17, 1–13. 10.1007/s40979-021-00072-y

[B156] Smith E. Williams-Jones B. (2012). Authorship and responsibility in health sciences research: a review of procedures for fairly allocating authorship in multi-author studies. Sci. Eng. Ethics 18, 199–212. 10.1007/s11948-011-9263-5 21312000

[B157] Soehartono A. M. Seki T. Khor K. A. Soehartono A. M. Seki T. Khor K. A. . (2022). Essential signals in publication trends and collaboration patterns in global Research Integrity and Research Ethics (RIRE). Scientometrics 127, 7487–7497. 10.1007/s11192-022-04400-y 35755633 PMC9206420

[B158] Solis Sánchez G. Alcalde Bezhold G. Alfonso Farnós I. (2023). Research ethics: from principles to practical aspects. An. Pediatr. 99, 195–202. 10.1016/j.anpede.2023.06.016 37598083

[B159] Sorokowski P. Groyecka A. Błaszczyński K. Frackowiak T. Kobylarek A. (2019). Registered reports as a method to increase credibility of science - experimental study among psychology students. J. Educ. Cult. Soc. 10, 67–75. 10.15503/jecs20192.67.75

[B160] Stahl B. Eke D. (2024). The ethics of ChatGPT - Exploring the ethical issues of an emerging technology. Int. J. Inf. Manag. 74:102700. 10.1016/j.ijinfomgt.2023.102700

[B161] Tahamtan I. Safipour Afshar A. Ahamdzadeh K. (2016). Factors affecting number of citations: a comprehensive review of the literature. Scientometrics 107, 1195–1225. 10.1007/s11192-016-1889-2

[B162] Tay L. Woo S. E. Hickman L. Booth B. M. D'Mello S. (2022). A conceptual framework for investigating and mitigating machine-learning measurement bias (MLMB) in psychological assessment. Adv. Methods Pract. Psychol. Sci. 5:25152459211061337. 10.1177/25152459211061337

[B163] Taylor L. (2017). What is data justice? The case for connecting digital rights and freedoms globally: Big Data Soc. 4:205395171773633. 10.1177/2053951717736335

[B164] Thorogood A. Knoppers B. M. (2017). Les comités d'éthique de la recherche peuvent-ils encourager le partage de données des essais cliniques ? Ethics Med. Public Health 3.

[B165] Tormo-Carbó G. Seguí-Mas E. Oltra V. (2018). Business ethics as a sustainability challenge: higher education implications. Sustainability 10:2717. 10.3390/su10082717

[B166] Tov W. Nai Z. L. S. (2019). “Cultural differences in subjective well-being,” in Subjective Well-Being and Life Satisfaction, ed. J. E. Maddux (London: Routledge), 50–73. 10.4324/9781351231879-3

[B167] Tzafestas S. (2018). Ethics and law in the internet of things world. Smart Cities 1, 98–120. 10.3390/smartcities101000623621718

[B168] Van Aert R. C. M. Niemeyer H. (2022). “Publication bias,” in Avoiding Questionable Research Practices in Applied Psychology, eds. W. O'Donohue, A. Masuda, and S. Lilienfeld (Cham: Springer Nature Switzerland AG), 213–242. 10.1007/978-3-031-04968-2_10

[B169] Verschuren P. Doorewaard H. (2010). Designing a ReseaRch PRoject.

[B170] Vesnic-Alujevic L. Nascimento S. Pólvora A. (2020). Societal and ethical impacts of artificial intelligence: critical notes on European policy frameworks. Telecommun. Policy 44:101961. 10.1016/j.telpol.2020.101961

[B171] Wenning C. J. (2001). Ethics in apiculture I. Am. Bee J. 141, 698–700.

[B172] Wikimedia Foundation (2024). Scopus. Available at: https://en.wikipedia.org/wiki/Scopus (accessed December 31, 2024).

[B173] Wilson E. Kenny A. Dickson-Swift V. (2018). Ethical challenges of community based participatory research: exploring researchers' experience. Int. J. Soc. Res. Methodol. 21. 10.1177/1049732317690721 29235941

[B174] Wolfram D. Wang P. Hembree A. Park H. (2020). Open peer review: promoting transparency in open science. Scientometrics 125, 1033–1051. 10.1007/s11192-020-03488-4

[B175] Wong P.-H. (2020). Democratizing algorithmic fairness. Philos. Technol. 33, 225–244. 10.1007/s13347-019-00355-w39533918

[B176] Woo S. E. LeBreton J. M. Keith M. Tay L. (2022). Bias, fairness, and validity in graduate-school admissions: a psychometric perspective. Perspect. Psychol. Sci. 18, 3–31. 10.1177/17456916211055374 35687736

[B177] Xiao W. S. (2021). The role of collectivism–individualism in attitudes toward compliance and psychological responses during the COVID-19 pandemic. Front. Psychol. 12:600826. 10.3389/fpsyg.2021.600826 34777076 PMC8581252

[B178] Yeoh M. P. Cazan A. M. Zaib S. Muss W. Jacic L. (2017). “Ethical and predatory publishing: experiences and perceptions of researchers,” in Bulletin of the Transilvania University of Brasov. Series VII, Social Sciences and Law 10.39149624

[B179] Zhang C. Liu L. Wang Y. (2021). Characterizing references from different disciplines: a perspective of citation content analysis. J. Informetr. 15:101134. 10.1016/j.joi.2021.101134

[B180] Zhang Y. Chen D. Cheng S. Liang Z. Yang L. Li Q. . (2023). Use of suboptimal control arms in randomized clinical trials of investigational cancer drugs in China, 2016-2021: an observational study. PLoS Med. 20:e1004319. 10.1371/journal.pmed.1004319 38085706 PMC10715645

[B181] Zwitter A. (2014). Big data ethics. Big Data Soc. 1, 1–6. 10.1177/2053951714559253

